# Genome-scale metabolic reconstruction of 7,302 human microorganisms for personalized medicine

**DOI:** 10.1038/s41587-022-01628-0

**Published:** 2023-01-19

**Authors:** Almut Heinken, Johannes Hertel, Geeta Acharya, Dmitry A. Ravcheev, Malgorzata Nyga, Onyedika Emmanuel Okpala, Marcus Hogan, Stefanía Magnúsdóttir, Filippo Martinelli, Bram Nap, German Preciat, Janaka N. Edirisinghe, Christopher S. Henry, Ronan M. T. Fleming, Ines Thiele

**Affiliations:** 1 School of Medicine, University of Galway, Galway, Ireland; 2 Ryan Institute, University of Galway, Galway, Ireland; 3 INSERM UMRS 1256, Nutrition, Genetics, and Environmental Risk Exposure (NGERE), University of Lorraine, Nancy, France; 4 Department of Psychiatry and Psychotherapy, University Medicine Greifswald, Greifswald, Germany; 5 Integrated BioBank of Luxembourg, Dudelange, Luxembourg; 6 University of Luxembourg, Esch-sur-Alzette, Luxembourg; 7 Czech University of Life Sciences Prague, Prague, Czech Republic; 8 Center for Molecular Medicine, University Medical Center Utrecht, Utrecht, the Netherlands; 9 Leiden Academic Centre for Drug Research, Leiden University, Leiden, the Netherlands; 10 Computation Institute, University of Chicago, Chicago, IL USA; 11 Mathematics and Computer Science Division, Argonne National Laboratory, Argonne, IL USA; 12 Division of Microbiology, University of Galway, Galway, Ireland; 13 APC Microbiome Ireland, Cork, Ireland

**Keywords:** Biochemical networks, Microbiome

## Abstract

The human microbiome influences the efficacy and safety of a wide variety of commonly prescribed drugs. Designing precision medicine approaches that incorporate microbial metabolism would require strain- and molecule-resolved, scalable computational modeling. Here, we extend our previous resource of genome-scale metabolic reconstructions of human gut microorganisms with a greatly expanded version. AGORA2 (assembly of gut organisms through reconstruction and analysis, version 2) accounts for 7,302 strains, includes strain-resolved drug degradation and biotransformation capabilities for 98 drugs, and was extensively curated based on comparative genomics and literature searches. The microbial reconstructions performed very well against three independently assembled experimental datasets with an accuracy of 0.72 to 0.84, surpassing other reconstruction resources and predicted known microbial drug transformations with an accuracy of 0.81. We demonstrate that AGORA2 enables personalized, strain-resolved modeling by predicting the drug conversion potential of the gut microbiomes from 616 patients with colorectal cancer and controls, which greatly varied between individuals and correlated with age, sex, body mass index and disease stages. AGORA2 serves as a knowledge base for the human microbiome and paves the way to personalized, predictive analysis of host–microbiome metabolic interactions.

## Abstract (web summary)

A resource of microbial metabolic models reveals how an individual’s gut microbiome influences drug metabolism.

## Main

Trillions of microorganisms inhabit the human gastrointestinal tract, with a high interindividual variation depending on factors, such as sex, age, ethnicity, lifestyle and health status^1^. The gut microbiota synthesizes bioactive metabolites, such as short-chain fatty acids, hormones and neurotransmitters^2^, and participates in the metabolism of commonly prescribed drugs^3^, resulting in drug inactivation, activation, detoxification or re-toxification^4^. Human gut microorganisms have been shown to metabolize 176 of 271 tested drugs^5^, with activity varying between individuals^6^. Consequently, precision medicine interventions that take diet, genetics and the microbiome into account have been proposed^7^. Predicting such personalized treatments would require detailed knowledge of the distribution of drug transformation reactions across human microbial taxa as well as the stoichiometry of these transformations.

A mechanistic systems biology approach that includes a detailed stoichiometric representation of metabolism is constraint-based reconstruction and analysis (COBRA)^8^. COBRA relies on genome-scale reconstructions of the target organisms which are often manually curated based on the available literature^8^. These reconstructions can be converted into predictive computational models through the application of condition-specific constraints^9^, including (meta-) omics and nutritional data, and linked together to interrogate strain-resolved, personalized microbiome models^10,11^. Hence, the COBRA approach is well suited for the exploration of metabolic human microbiome cometabolism^12,13^. To facilitate the genome-scale reconstruction of the thousands of known species inhabiting humans^14^, semiautomated reconstruction tools, such as CarveMe^15^, MetaGEM^16^, MIGRENE^17^ and gapseq^18^, have been published. Despite their many advantages, these tools provide limited support for curation against manually refined genome annotations and experimental data from peer-reviewed literature. Both are crucial for the inclusion of not yet routinely annotated species-specific pathways (for example, drug metabolism)^9^. To overcome these limitations, we have developed a semiautomated curation pipeline guided by manually assembled comparative genomic analyses and experimental data^19^, which previously enabled the generation of AGORA, a resource of 773 genome-scale reconstructions of human gut microorganism strains, representing 605 species and 14 phyla^20^.

Here, we present an expansion in scope and coverage of AGORA, called AGORA2, consisting of microbial reconstructions for 7,302 strains, 1,738 species and 25 phyla. AGORA2 summarizes the knowledge and experimental data obtained through manual comparative genomics analyses and literature and textbook review, and demonstrates high accuracy against three independently collected experimental datasets. AGORA2 has been expanded by manually formulated molecule- and strain-resolved drug biotransformation and degradation reactions covering over 5,000 strains, 98 drugs and 15 enzymes, some of which were validated against independent experimental data. The AGORA2 reconstructions are fully compatible with the generic^21^ and the organ-resolved, sex-specific, whole-body human metabolic reconstructions^22^. We demonstrate the use of AGORA2 for the prediction of personalized gut microbial drug metabolism for a cohort of 616 individuals. Taken together, the AGORA2 reconstructions can be used independently or together for investigating microbial metabolism and host–microbiota cometabolism in silico.

## Results

### Data-driven reconstruction of diverse human microorganisms

To build the reconstructions of the 7,302 gut microbial strains in the AGORA2 compendium (Supplementary Table 1), we substantially revised and expanded (Methods) a previously developed^20^ data-driven reconstruction refinement pipeline, deemed DEMETER (Data-drivEn METabolic nEtwork Refinement)^19^. Overall, the DEMETER workflow consists of data collection, data integration, draft reconstruction generation, and translation of reactions and metabolites into the Virtual Metabolic Human (VMH)^23^ name space, and simultaneous iterative refinement, gap-filling and debugging^19^. Reconstruction refinement follows standard operating procedures for generating high-quality reconstructions^9^ and is continuously verified through a test suite^19^ (Supplementary Table 2 and Supplementary Note 2).

**Fig. 1:**
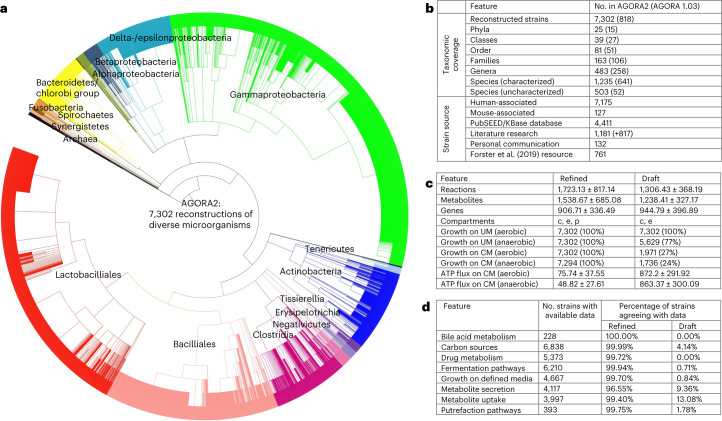
Features of AGORA2. **a**, Taxonomic coverage and sources of reconstructed strains**. b**, Taxonomic distribution of the included 7,302 strains**. c**, Features of the AGORA2 reconstructions and KBase draft reconstructions. c, cytosol; e, extracellular space; p, periplasm. Growth rates on Western diet (WD) and unlimited medium (UM) are given in  h^−1^ (Methods). ATP production potential on WD is given in mmol per g_dry weight_ per h. Shown are averages across all models ±standard deviations. **d**, Number of reconstructions with available positive findings from comparative genomics and literature, and percentage of curated and draft reconstructions agreeing with the findings for the respective organism. N/A, not applicable as the pathway was absent in draft reconstructions. CM, chemically defined medium.

After expanding the taxonomic coverage (Fig. 1a,b, Supplementary Table 1 and Supplementary Note 1) and retrieving the corresponding genome sequences, we generated automated draft reconstructions through the online platform KBase^24^, which were subsequently refined and expanded through the DEMETER pipeline^19^ (Methods). As a lack of accurate genome annotations is a source of uncertainty in the predictive potential of genome-scale reconstructions^25^, we manually validated and improved the annotations of 446 gene functions across 35 metabolic subsystems for 5,438 of 7,302 (74%) genomes using PubSEED^26^ (Supplementary Table 3a–d). To further ensure accurate representation of species-specific metabolic capabilities, we performed an extensive, manual literature search spanning 732 peer-reviewed papers and two microbial reference textbooks, yielding information for 6,971 of 7,302 strains (95%) (Methods). For the remaining 331 strains, either no experimental data were available or all biochemical tests reported in the literature were negative. The performed extensive refinement driven by the collected data resulted on average in the addition of 685.72 (standard deviation: ±620.83) reactions and removal of 685.72 (standard deviation: ±620.83) reactions per reconstruction (Supplementary Fig. 1). The biomass reactions provided in the draft reconstructions were curated, and reactions were placed in a periplasm compartment where appropriate (Supplementary Note 3). Moreover, we retrieved the metabolic structures for 1,838 of 3,613 (51%) metabolites and provide atom–atom mapping for 5,583 of the overall 8,637 (65%) enzymatic and transport reactions captured across AGORA2 (Methods). Owing to these extensive curation efforts, the metabolic models derived from the refined reconstructions showed a clear improvement in their predictive potential over models derived from the KBase draft reconstructions (Fig. 1c,d and Supplementary Note 2). As an additional assessment of reconstruction quality, we generated an unbiased quality control report for all reconstructions (Methods) resulting in an average score of 73%.

**Fig. 2:**
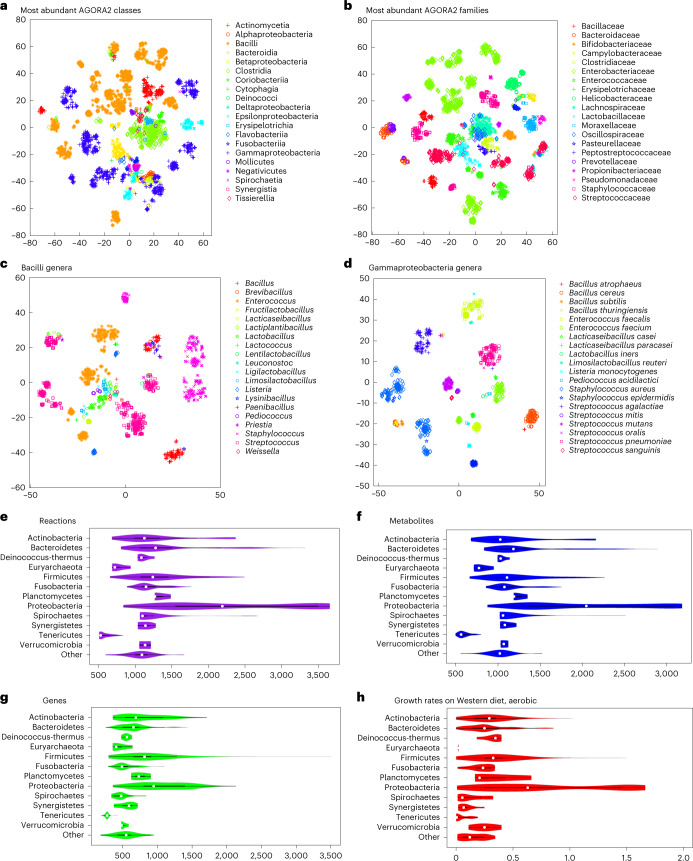
Taxonomically related strains are similar in their AGORA2 reconstruction content. **a**–**d**, Clustering through *t*-SNE^52^ of reaction presence across all pathways per reconstruction. Coordinates were statistically different across taxonomic units (Kruskal–Wallis test, *P* = 0.0001 in all cases). **a**, Members of the largest classes. **b**, Members of the largest families. **c**, Members of the Bacilli class by genus. **d**, Members of the Gammaproteobacteria class by genus. **e**–**h**, Features of all AGORA2 reconstructions across phyla: **e**, Number of reactions. **f**, Number of metabolites. **g**, Number of genes. **h**, growth rate in h^−1^ on aerobic Western diet.

We then clustered the content of the AGORA2 reconstructions by taxonomic distribution. Overall, AGORA2 reflects the diversity of the captured strains as they clustered by class and family according to their reaction coverage (Fig. 2a,b, Supplementary Fig. 3a and Supplementary Note 4). Several genera in the Bacilli and Gammaproteobacteria classes formed subgroups illustrating important metabolic differences between them (Fig. 2c,d, Supplementary Fig. 2a,b and Supplementary Note 4; Kruskal–Wallis test: *P* = 0.0001). Cross-phylum metabolic differences also translated to differences in reconstruction sizes and predicted growth rates (Fig. 2e–h) and in their potential to consume and secrete metabolites (Supplementary Fig. 3a,b). Taken together, the models derived from AGORA2 capture taxon-specific metabolic traits of the reconstructed microorganisms.

### AGORA2 is predictive against three independent datasets

While automated draft reconstructions can be rapidly generated, they still require subsequent curation efforts to be predictive^27^. Several (semi)automated reconstruction tools bridge the gap between automated draft and fully manually curated reconstructions including CarveMe^15^, gapseq^18^ and MIGRENE^17^. To further access the quality of AGORA2 and the DEMETER pipeline, we compared AGORA2’s predictive potential and model properties with other resources of microbial genome-scale reconstructions. For this purpose, we retrieved 8,075 reconstructions built through gapseq^18^, 1,333 reconstructions built through MIGRENE, deemed MAGMA^17^, as well as 72 manually curated genome-scale reconstructions deposited in the BiGG database^28^. Additionally, we built CarveMe^15^ reconstructions for 7,279 AGORA2 strains and gapseq^18^ reconstructions for a subset of 1,767 AGORA2 strains (Methods).

**Fig. 3:**
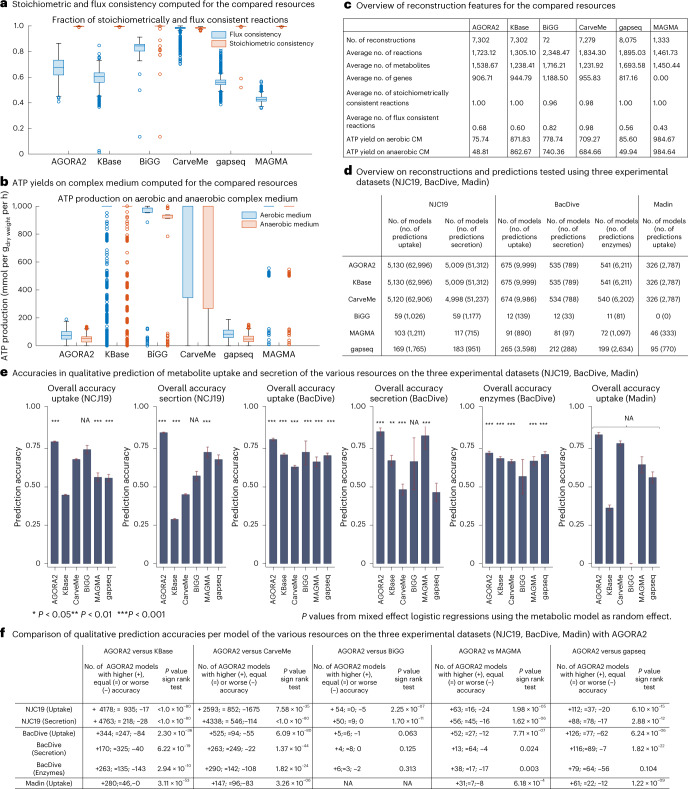
Comparison of AGORA2-refined reconstructions, draft reconstructions and three other reconstructions resources. Compared were the 7,302 AGORA2 and KBase draft reconstructions, 72 manually curated reconstructions from the BiGG database^28^, 5,587 reconstructions built through CarveMe^15^, 8,075 reconstructions built through gapseq^18^ and 1,333 MAGMA reconstructions^17^. **a**, Fraction of reactions that are stoichiometrically and flux consistent as defined in ref. ^29^ for each model derived from the five compared resources. Exchange and demand reactions, which are stoichiometrically inconsistent by definition, were excluded. **b**, Aerobic and anaerobic ATP production on complex medium (mmol per g_dry weight_ per h) by each model derived from the five compared resources. **c**, Overview of reconstruction properties for the compared resources. **d**, Overview of number of models and number of predictions tested in validating AGORA2, KBase, BiGG, CarveMe, gapseq and MAGMA against three independent experimental datasets^30,32,33^. **e**, Bar plots with 95% confidence intervals of overall accuracies of the five resources in predicting uptake and secretion in the three experimental datasets. Significance of prediction accuracy was determined by mixed effect logistic regressions using the metabolic model as random effect variable to account for the statistical dependence of predictions stemming from the same model. NA indicates a missing *P* value due to empty categories (for example, no true negatives detected). **f**, Comparison of accuracies per model of the various resources on the three experimental datasets. *P* values were derived by sign rank tests.

For an unbiased assessment of reconstruction quality, we first determined the fraction of flux consistent reactions^29^ in each resource. Only the manually curated reconstructions from BiGG and reconstructions built through CarveMe had a higher fraction of flux consistent reactions than AGORA2 (Fig. 3a,b; *P* < 1 × 10^−30^, Wilcoxon rank-sum test). Note that our reconstructions represent knowledge bases; thus, if genetic or biochemical evidence exists for a gene or reaction, it will be included in the reconstruction. In contrast, CarveMe by design removes all flux inconsistent reactions from a metabolic reconstruction^15^. Compared with the KBase draft reconstructions, AGORA2 had a significantly higher percentage of flux consistent reactions despite being larger in metabolic content, as well as a significantly higher flux consistency than gapseq and MAGMA (Fig. 3a,c; *P* < 1 × 10^−30^, Wilcoxon rank-sum test). It was also observed that all resources except AGORA2 and gapseq produced very high amounts of ATP (up to 1,000 mmol g_dry weight_^−1^ h^−1^) on the complex medium for at least a subset of models (Fig. 3b,c). Hence, in these models, the ATP production flux was only limited by the upper bounds on reactions, which generally indicates the existence of futile cycles^9^.

The most crucial aspect of a genome-scale reconstruction is its accuracy in capturing known biochemical or physiological traits of the target organism^9^, that is, its potential to make biologically plausible predictions. Hence, we set out to determine the predictive potential of AGORA2. For an unbiased assessment, we retrieved organism-specific experimental data from three separate sources (Methods). First, we retrieved species-level positive and negative metabolite uptake and secretion data for 455 species (5,319 strains) in AGORA2 from the NJC19 resource^30^. Note that a precursor of NJC19, NJS16 (ref. ^31^), containing only positive data, had been used to refine AGORA2. Next, we mapped species-level positive metabolite uptake data, retrieved from Madin et al.^32^, for 185 species (328 strains) in AGORA2 (‘Madin’ data). Finally, we retrieved strain-resolved positive and negative metabolite uptake and secretion data for 676 AGORA2 strains as well as positive and negative enzyme activity data for 881 AGORA2 strains from the BacDive database^33^. Neither the Madin dataset nor BacDive had been used during the refinement of AGORA2. For metabolite uptake and secretion, the AGORA2 reconstructions captured the known capabilities of the target organisms very well (overall accuracy against NJC19, BacDive and Madin of 0.82, 0.81 and 0.84, respectively; Fig. 3e and Supplementary Table 4d). For enzyme activity, a slightly lower accuracy of 0.72 was achieved (Fig. 3e and Supplementary Table 4d). AGORA2 had a lower specificity than the other resources on NJC19. However, the majority of observed false positives in AGORA2 concerned glutamate uptake in *Escherichia coli* (Supplementary Table 4c), which was a negative finding in the NJC19 dataset based on a report for a single *E. coli* strain.

We then compared the predictive potential of AGORA2 with the other four resources where possible. Of the 7,302 reconstructed AGORA2 strains, 7,279 had been reconstructed through CarveMe, 451 overlapped with reconstructions built through gapseq and 60 overlapped with reconstructed strains available at the BiGG database (Supplementary Table 4a). No strains overlapped with MAGMA as it consists of pan-species reconstructions built from metagenome-assembled genomes^17^, but 216 reconstructions could be mapped at the species level (Supplementary Table 4a). For the four resources and for each dataset, we then computed the predictive potential for the organisms overlapping with AGORA2 (Fig. 3d–f and Supplementary Table 4b–d). While MAGMA and AGORA2 achieved significant prediction accuracies for secretion and uptake on the NJC19 and the BacDive datasets, KBase failed to perform better than chance for metabolite uptake and secretion in NJC19, and CarveMe failed to predict significantly secretion in the NJC19 dataset (Fig. 3e and Supplementary Table 4d). The gaqseq reconstructions built in the present study for the subset of AGORA2 strains performed comparably to the set of gapseq reconstructions that had been published by the authors^18^ (Supplementary Table 4b).

To compare the performance of AGORA2 with KBase, CarveMe, gapseq, BiGG and MAGMA directly, we calculated the accuracy per model separately for uptake and secretion. We then compared the accuracies on models in the overlap of AGORA2 and each resource via a nonparametric sign rank test. AGORA2 was significantly better than all other methods on all three datasets, except for BiGG on the BacDive data, where the overlap in models was too small to achieve sufficient statistical power, and gapseq on the BacDive enzyme data where it performed comparably to AGORA2 (71% versus 72%; Fig. 3e,f).

Taken together, the AGORA2 reconstructions capture the known traits of the respective organisms very well, surpassing other semiautomatedly generated reconstructions and being comparable to manually curated reconstructions. These results demonstrate the value of the extensive curation efforts refinement, guided by species–species experimental data, performed during the development of AGORA2 as outlined above. Accordingly, AGORA2 performed particularly well for metabolite uptake and secretion data, which require curation based on experimental data, compared with enzyme activity data, which can be curated based on genome annotations. Remaining false positive and false negative predictions (Supplementary Table 4c) will be addressed in future efforts following the iterative curation philosophy^9^. Flux inconsistent reactions, indicating they contain dead-end metabolites^29^, may serve as the starting point for gap-filling efforts, thereby enabling biological discovery^34^.

### Microbial drug metabolism guided by genome and bibliome

**Fig. 4:**
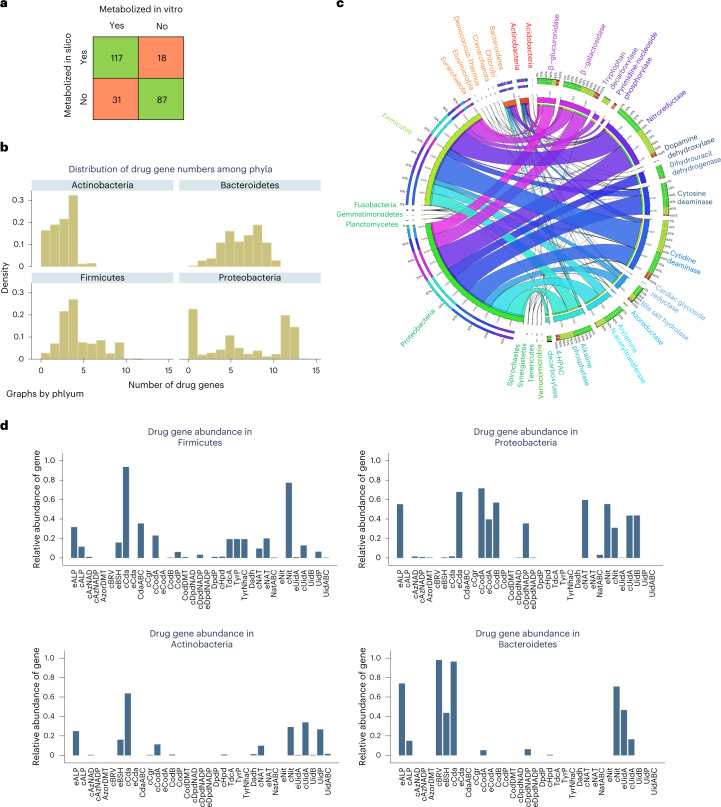
Overview of reconstructed drugs and annotated drug enzymes present in AGORA2. **a**, Overlap between independent, experimentally demonstrated activity of drug-metabolizing enzymes and predictions by models derived from the AGORA2 reconstructions for 253 drug–microbe pairs (Supplementary Table 7). **b**, Distribution of the number of strains carrying each drug enzyme over the 14 analyzed phyla. **c**, Fraction of strains carrying each gene encoding drug enzymes or transport proteins in the four main phyla in the human microbiome. **d**, Distribution of the number of drug genes per strain for the four main phyla. For the list of abbreviations, see Supplementary Table 3b.

Microorganisms can directly or indirectly influence drug activity and toxicity through degradation (for example, hydrolysis), and biotransformation (for example, reduction)^3,4^ . However, drug metabolism is only captured to a limited extent by genome annotation pipelines and no systematic comparative genomic analysis of drug-metabolizing enzymes has previously been performed. Hence, microbial drug transformations are not yet captured by any existing genome-scale reconstruction resources. To fill this gap, we performed an extensive, manual comparative genomic analysis for 25 drug genes, encoding for 15 enzymes shown to directly or indirectly affect drug metabolism (Supplementary Table 5a), their subcellular locations and 12 genes encoding for drug transporters (Supplementary Table 3b). All 5,438 analyzed strains carried at least one drug-metabolizing enzyme (Supplementary Table 3c). As these enzymes are also involved in central metabolism, for example, nucleoside metabolism, this high coverage was expected. We then carried out a thorough literature and database review of metabolite structures, formulas and charges for 98 frequently prescribed drugs belonging to ten drug groups and 32 subgroups (Supplementary Table 5b). We formulated 1,440 drug-related reactions containing 363 metabolites (Supplementary Table 6a,b) and added, on average, 188 drug-related reactions and 111 metabolites to the reconstructions depending on the genomic evidence. We validated, with an accuracy of 0.81 (sensitivity: 0.87, specificity: 0.74, Fisher’s exact test: *P* = 2.01 × 10^−23^, mixed effect logistic regression accounting for stochastic dependencies from predictions stemming from the same model: *P* = 1.209 × 10^−07^), the drug-metabolizing predictions against independent published experimental data for 253 drug–microbe pairs (Supplementary Table 7 and Fig. 4a). The 18 false positive predictions may indicate nonfunctional genes or regulatory mechanisms, whereas the 31 false negative predictions could be due to incompleteness of genomes or nonorthologous displacement in complete genomes, or a currently unaccounted for homolog encoding the reaction.

### Taxonomic distribution of drug-metabolizing capabilities

We next analyzed the taxonomic distribution of the annotated drug and transport genes (Fig. 4b–d and Supplementary Table 3c). At least one strain in each of the 14 analyzed phyla encoded for genes involved in drug metabolism (Fig. 4f). The most widespread drug-metabolizing enzymes were cytidine deaminase and nitroreductase, which were found in 12 and 13 phyla, respectively (Supplementary Fig. 7a,b). Another central metabolic enzyme, the pyrimidine-nucleoside phosphorylase, was also widely distributed, but the monophyletic branch specific for the metabolism of brivudine and sorivudine^35^ was only found in the Bacteroidetes phylum (Fig. 4c,d and Supplementary Fig. 7c). Many drugs are detoxified by the liver through the addition of glucuronic acid, a modification that is reversed by microbial β-glucuronidase^4^. This enzyme was in >99% of analyzed *E. coli* strains and was also widely distributed across Bacteroidetes and Firmicutes strains (Fig. 4c,d and Supplementary Fig. 7d), consistent with previous analyses^36^. *E. coli* was the species most enriched in drug metabolism, with >99% of all analyzed strains carrying seven to ten drug enzymes (Supplementary Table 3c). Taken together, drug-metabolizing enzymes and transporters, are widely distributed, but important phyla-specific and strain-specific differences exist. To elucidate the potential benefits that these drug-metabolizing capabilities could confer to the microorganisms, we computed the strain-specific energy, carbon and nitrogen yields of drug degradation. This analysis revealed that many strains spread across phyla were capable of using drugs as a source of energy, carbon and/or nitrogen (Supplementary Fig. 4 and Supplementary Table 8).

### Personalized modeling of drug-metabolizing capacities

**Fig. 5:**
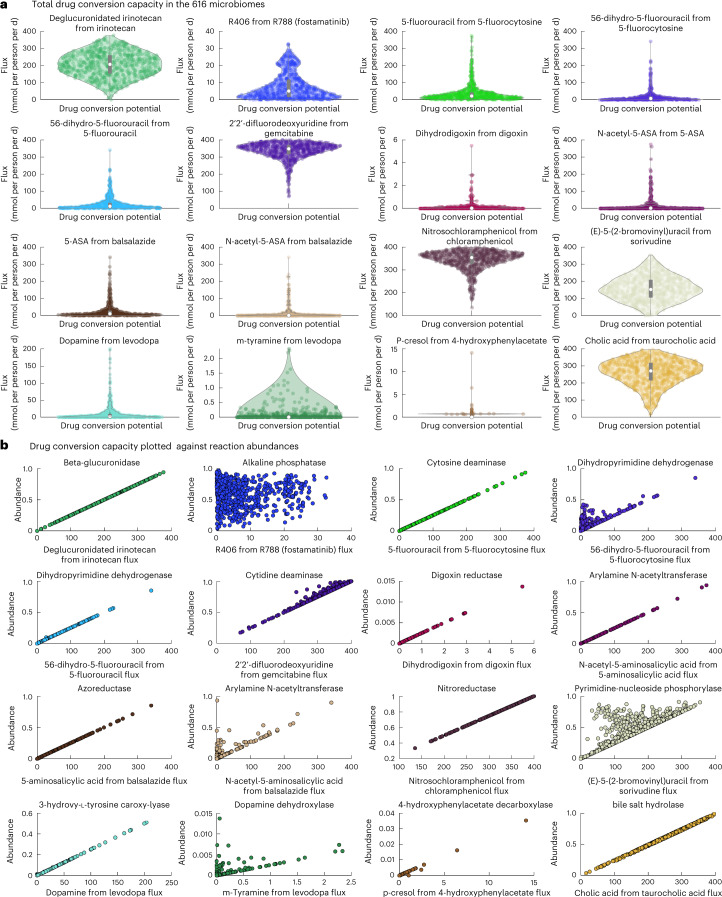
Drug conversion capacity of 616 microbiomes. **a**, Drug conversion potential in the microbiomes of 365 Japanese patients with CRC and 251 controls on the Average Japanese Diet. The violin plots show the distribution of drug metabolite flux in mmol per person per d. **b**, Drug conversion potential (mmol per person per d) plotted against the total relative abundance of the reaction producing the shown drug metabolite in the 616 microbiomes. See Supplementary Table 5a for a description of each drug-metabolizing enzyme.

As human microorganisms do not exist in isolation, we addressed the important question of how the total drug-metabolizing capacities may differ between individual gut microbiomes. A previously developed community modeling framework^10^ allows for the scalable, tractable computation of community-wide metabolic capabilities as well as organism-resolved contributions to fecal metabolite levels^37^. We used a metagenomic dataset from a Japanese cohort of 365 patients with colorectal cancer (CRC) and 251 healthy controls^38^ that had previously allowed us to interrogate the metabolic capabilities of each gut microbiome and validate the fluxes against metabolomic data^37^. A total of 97% of the named species could be mapped onto AGORA2 (compared with 72% for AGORA). For each individual’s gut microbiome, we built and interrogated a community model (Methods), resulting in the prediction of total drug-metabolizing potential (Fig. 5a and Supplementary Table 9). For some enzymes, for example, dihydropyrimidine dehydrogenase and dopamine dehydroxylase, the drug conversion potential only showed limited correlation with the total abundance of the corresponding drug-metabolizing reactions, indicating flux-limiting metabolic bottlenecks (Fig. 5b). Analyzing such bottlenecks would require the simulation of enzymatic functions in their metabolic context. Shadow price analysis (Methods) revealed that, in two-step reactions, such as levodopa degradation to m-tyramine, the drug conversion potential for the second step was limited by the species abundance carrying out the first step (Supplementary Note 5, Supplementary Fig. 6 and Supplementary Table 10). Levodopa degradation is known to be a two-step pathway carried out by different species^39^ (Supplementary Fig. 6).

While most drugs could be qualitatively metabolized in silico by at least 95% of the microbiomes, only 53% of the microbiomes presented the capacity to metabolize digoxin, and levodopa could be metabolized by 86% of the investigated microbiomes into dopamine and by 46% into m-tyramine (Fig. 5a). Both digoxin transformation and the second step of levodopa degradation strictly depended on the presence of *Eggerthella lenta* (Supplementary Fig. 8), and are known to reduce bioavailability of the drugs^4,39^. Moreover, while all but three microbiomes could activate the anti-inflammatory bowel disease (IBD) prodrug balsalazide through the azoreductase activity, the highest secretion flux of the active form of balsalazide (5-aminosalicylic acid) achieved by any microbiome was 339.81 mmol d^−1^ per person, while the average was 25.47 ± 40.84 mmol d^−1^ per person (Fig. 5a). This variation may be of high clinical relevance, as it indicates that not all microbiomes can equally activate balsalazide. As a sensitivity analysis, we recomputed drug-metabolizing capacities using an average European diet instead of the Japanese diet and found that the drug-metabolizing capacities were virtually unaltered for all drugs and, hence, highly robust towards diet constraints (Supplementary Fig. 7).

### Microbiome-level fluxes are sensitive to clinical parameters

**Fig. 6:**
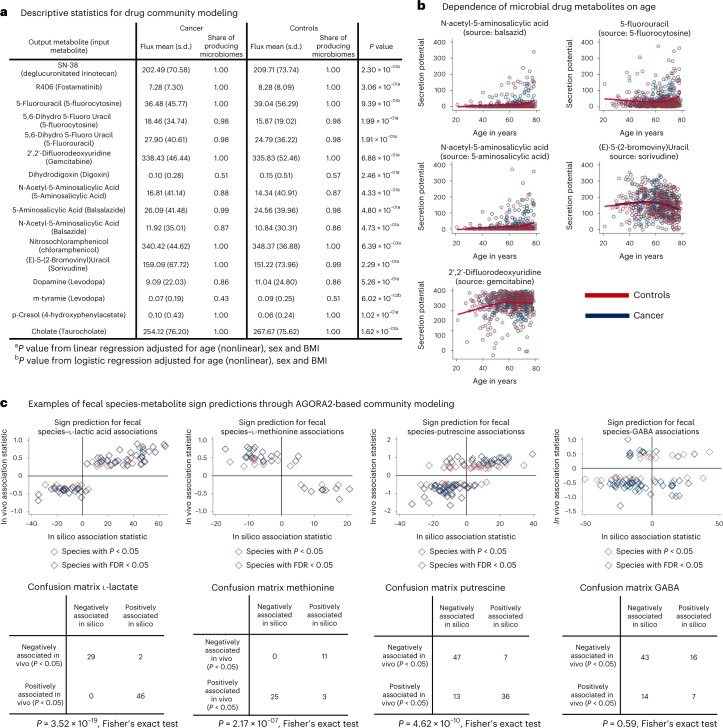
Descriptive statistics for the modeled drug metabolites and fecal species–metabolite associations. **a**, Overview of descriptive statistics for the modeled drug metabolites. **b**, Scatter plots (red, controls; blue, cancer) of various drug metabolites in dependence on age with nonlinear regression lines for cases and controls. Regression lines were estimated with restricted cubic splines. All regression models had *P* < 0.0001 (FDR < 0.05) and regression coefficients were virtually the same for cases and controls. **c**, Fecal species metabolite sign prediction for l-lactic acid, l-methionine and gamma-aminobutyrate. Upper panel represents scatter plots of in silico change in microbial community net secretion flux derived from community modeling against the change in measured fecal concentration in dependence on microbial species presence. Each dot represents one microbial species having an effect on metabolite concentration with at least *P* < 0.05. Lower panel depicts the confusion matrix of sign prediction through in silico modeling. *P* values derived from Fisher’s exact test should be treated with care due to species–species and metabolite–metabolite interdependencies.

Next, we investigated whether drug-metabolizing capacities were associated with CRC. For none of the drugs, including cancer drugs, neither qualitative nor quantitative differences in drug-metabolizing capacities were found after correction for multiple testing, despite the reported enrichment in 29 species in CRC metagenomes^40^. On a nominal level (*P* < 0.05), nitrosochloramphenicol was increased in cancer cases (Fig. 6a). Nonetheless, drastic individual differences in drug-metabolizing potential, regardless of disease status, due to distinct microbiota composition existed (Fig. 5a).

Lastly, we investigated the statistical association pattern of age, sex and body mass index (BMI) to the drug-metabolizing capacities of the microbiome (Fig. 6b and Supplementary Fig. 9). Five predicted secretion potentials of drug metabolites were clearly associated with age (Fig. 6b), although the effect sizes were small to medium (explained variances <8%). For example, the conversion of sorivudine into a toxic byproduct showed a nonlinear association with age, where secretion capacities declined from 60 yr on (Fig. 6b; *R*^2^ = 0.047, *P* = 7.17 × 10^−06^). Women had significantly higher taurocholate metabolizing capability, and slightly, but significantly, lower conversion potential of the chemotherapy drug gemcitabine (Supplementary Fig. 9a). In conclusion, our analysis enabled investigation into clinical parameters that were associated with drug-metabolizing capacities of the gut microbiome.

### Community models predict species–metabolite associations

As a last step of validation, we tested whether AGORA2-based community modeling is capable of predicting the sign of statistical associations between microbial species presence and fecal metabolite concentrations in the CRC sample, following procedures established before^41^. We calculated the fecal net secretion rate for 52 AGORA metabolites (Methods), for which fecal metabolomics data from the same Japanese cohort were available^38^. As these metabolomics data were not used in constructing the AGORA2-based community models, this procedure represents an independent validation.

After correction for multiple testing, AGORA2-based community modeling was predictive for the sign of significant species–metabolite associations in 24 of 52 metabolites (Fig. 6c and Supplementary Table 11) with *P* < 0.05 and 19 with false discovery rate (FDR) < 0.05. Particularly well covered were amino acids and known fermentation products (for example, l-lactate, butyrate), as well as amines (Supplementary Table 11). Notably, for certain metabolites, for example, methionine (Fig. 6c), in vivo association statistics were consistently inverse to the corresponding in silico association statistics. These latter results may correspond to net uptake of the metabolites by the microbial community. The nonsignificant sign prediction, as exemplarily depicted in Fig. 6c for gamma-aminobutyrate, can have multiple reasons, ranging from host factors dominating the variation in fecal concentration to incomplete community models or missing confounders in the statistical models, leading to false positive in vivo associations. In conclusion, AGORA2-based community models could predict the direction of species metabolite associations for a broad range of metabolites, highlighting the models’ predictive natures.

## Discussion

Here, we introduced AGORA2, a resource of 7,302 genome-scale reconstructions for human-associated microorganisms with coverage, scope and curation effort that is, to our knowledge, unprecedented. AGORA2 follows the quality standards developed by the systems biology research community^9,42^, accurately captures biochemical and physiological traits of the target organisms, surpassing other reconstruction resources, and includes manually refined, strain-resolved drug-metabolizing capabilities. It enables personalized modeling of human microbial metabolism through a dedicated computational pipeline^10^, which had recently been improved in terms of computational efficiency and implemented features^43^. Hence, personalized microbiome modeling using AGORA2 can be performed in a reasonable timeframe on a standard personal computer (Methods).

Computational modeling of microbial consortia is increasingly recognized as a complementary method to in vitro and in vivo experiments and can generate experimentally testable hypotheses^13,44^. Our knowledge about gut microorganisms remains limited and, thus, any in silico reconstruction will be inherently incomplete and require regular updates^45^. For instance, a recent study has found that 176 of 271 tested drugs could be metabolized by human bacteria, and, for a subset of these drugs, transformations could be linked to specific gene functions^5^. Through future comparative genomics and metabolite and reaction formulation efforts, AGORA2 may be expanded by these drug transformations to further broaden its coverage of prescription drug metabolism. As AGORA2 uses the same metabolite and reaction nomenclature^23^ as the human metabolic reconstruction^21^ and the whole-body metabolic reconstructions^22^, it could be used to predict overall host–microbiome cometabolism as well as their potential contribution to human organ-level metabolism^22^.

To date, AGORA has enabled nearly 50 studies that modeled microbe–microbe, host–microbe and microbiome interactions^46^, and, together with available software tools^10,47^, contributed substantially to recent advances in size and scope of constraint-based modeling of multispecies interactions^46^. However, AGORA was to an extent hampered by its limited taxonomic coverage, which mainly included the Westernized gut microbiome^20^. In contrast, AGORA2 also captures microorganisms commonly found in non-Westernized microbiomes as well as in skin, oral and vaginal microbiomes; includes many uncultured microorganisms; and has a high overlap with species reported in several resources of metagenome-assembled genomes (Supplementary Note 1). Together, this extension increases the prediction fidelity of microbiome-level models included for nongut and non-Westernized microbiomes.

We reported associations between CRC patient-specific microbial drug conversion capabilities and clinical parameters, such as age and BMI (Fig. 6). The example of balsalazide, an anti-inflammatory drug utilized in treating IBD, showcases how AGORA2 could be used to inform clinical research, and potentially facilitate the personalization of treatment. Balsalazide has high number needed to treat (NNT) metrics for inducing remission (NNT: 10) and maintenance (NNT: 6) in ulcerative colitits^48^, indicating that most patients do not profit from the drug. Consistently, the balsalazide activation potential varied strongly in the investigated CRC cohort microbiomes (Fig. 5a), indicating that not all individuals would profit equally from balsalazide treatment. Consequently, we propose that AGORA2 in conjunction with metagenomics could predict the stratification of patients with IBD into balsalazide responders and nonresponders, which could then be validated in follow-up clinical trials. The finding that drug-metabolizing capabilities were associated with age groups, BMI and sex (Fig. 6b and Supplementary Fig. 9) demonstrates that AGORA2 in conjunction with community modeling can be utilized in large epidemiological cohort studies to link predicted metabolic fluxes with clinical parameters, thereby opening new research possibilities to understand the role of the microbiome in modifying health risk and contributing to adverse health outcomes. Finally, AGORA2-based community models were able to predict the direction of species–metabolite associations for a range of metabolites (Fig. 6c), demonstrating utility in delivering valid in silico markers of the microbiome’s metabolic traits.

Taken together, we present a resource of genome-scale metabolic reconstructions, AGORA2, which accurately captures organism-specific capabilities and can be used to build predictive personalized microbiome models. AGORA2 and all tools and scripts used in this study are freely available to the research community. We expect that similar to its predecessor AGORA2 will be of great interest to the microbiome and constraint-based modeling communities, with an even broader range of potential applications^46^. As a unique feature, AGORA2 captures strain-resolved microbial drug metabolism. Predicting drug response to realistic drug concentrations will require hybrid modeling approaches, for example, integrating constrained-based modeling with physiological-based pharmacokinetic modeling^49,50^. Using a constrained-based model of organ-resolved whole-body metabolism integrated with models of the gut microbiome^22^, and using such hybrid modeling approaches, dietary supplements, probiotics or microbiome-targeted interventions, which have been shown to attenuate side effects of drugs^4^, could be predicted and validated^49^. Hence, AGORA2 paves the way for an integrative, multi-scale modeling approach that may enable in silico clinical trials^49,51^ and contribute to precision medicine.

## Methods

### Selection of newly reconstructed organisms and retrieval of whole-genome sequences

First, we retrieved 4,185 genomes of human gut-associated strains that were available on PubSEED^53^ (Supplementary Note 6). To expand the species coverage, we performed an extensive literature search of species isolated from or detected in the human microbiome with available whole-genome sequences (Supplementary Table 1). This search led to the addition of a further 1,324 strains, which included 127 genomes of mouse-associated strains. The corresponding whole-genome sequences were retrieved in FASTA format from the National Center for Biotechnology Information (NCBI) FTP site (ftp://ftp.ncbi.nlm.nih.gov/). Moreover, we included 26 genomes of *Eggerthella lenta* strains^54^ available at https://www.ncbi.nlm.nih.gov/bioproject/PRJNA412637. Finally, we retrieved 761 human microbial genomes from the Human Gastrointestinal Bacteria Culture Collection^55^ in FASTQ format from https://www.ebi.ac.uk/ena/data/view/PRJEB23845 and https://www.ebi.ac.uk/ena/data/view/PRJEB10915. Together with AGORA1.03, which was obtained from the VMH^23^, these combined efforts resulted in 7,302 strains and 1,738 species included in AGORA2.

### Manual refinement of metabolic pathways and gene annotations through comparative genomics

Of the 7,302 analyzed strains, 5,438 bacterial strains and three archaeal strains were present in the PubSEED resource^53,56^ (Supplementary Note 6) and could be re-annotated for their metabolic functions through comparative genomics. A total of 34 metabolic subsystems that had been reconstructed previously for a smaller subset of gut microbial strains^20,57–60^, as well as a newly created drug metabolism subsystem, were considered for the analysis (Supplementary Table 3a for a comprehensive list of subsystems). All subsystems are available at the PubSEED website.

#### Curation of subsystems

For annotation of the genes in each subsystem, the PubSEED platform was used^53^. Functional roles for each subsystem were annotated based on the (1) prescribed functional role for the protein, (2) sequence similarities of the protein to proteins with previously confirmed functional roles and (3) genomic context (Supplementary Note 7).

#### Metabolic pathways considerations for comparative genomics analysis

Absence of gene(s) for one or more enzymes in a pathway may result in blocked reactions in a metabolic reconstruction. To avoid this, we estimated the completeness of metabolic pathways during the genome annotation. For each potentially synthesized metabolite, all the biosynthetic pathways were collected in agreement with the KEGG PATHWAY resource^61^ and genes of the subsystem were attributed to corresponding steps of the metabolic pathways. Absence of the consequent reactions was determined as a gap. Only pathways with no more than two gaps with gap length of no more than one step (Supplementary Note 8) were further gap-filled and used for generation of reactions.

#### Sequence-based gap-filling

For the gapped pathways, the bidirectional best-hit (BBH) method^62^ was used: (1) The gene corresponding to the gap and present in the genome for the related organisms (belonging to the same species, genus, or family) was used as a query for a BLAST search in the genome with the gap. (2) Possible BBHs were defined as homologs for that alignment with the query protein having an e-value < −50 and protein identity ≥50%. (3) For each possible BBH, the reverse search was done for the genome that was a source of the query protein. (4) If the query protein and its best homolog in the analyzed genome formed a BBH pair, the gap was filled. (5) A similar genomic context for the query protein and its ortholog was considered as an additional confirmation for orthology of the identified BBH pair.

#### Annotation of the drug metabolic genes

To annotate drug-metabolizing genes, we used the following pipeline. (1) Identify genes known to encode for drug-metabolizing enzymes in a range of microbial organisms, from the scientific literature (Supplementary Table 5a). (2) Using the amino acid sequences of these known drug-metabolizing genes as queries, we performed a BLAST search for every analyzed genome. (3) The resulting best BLAST hit was then used as a query for the BLAST search in the genome having a known drug-metabolizing gene to confirm that the known protein sequence and its best BLAST hit form a pair of BBHs. (4) All BBHs were used for the construction of a rooted maximal-likelihood tree. (5) All previously known proteins were mapped onto the tree, and all monophyletic branches containing known drug-metabolizing enzymes were determined (Supplementary Fig. 10). (6) All annotated proteins in these branches were considered as orthologs of the known drug-metabolizing proteins. All the proteins not being in branches with known drug-metabolizing proteins were considered as proteins with other functions and were excluded from further analysis. Subsequently, a tree was constructed again for orthologs of the known drug-metabolizing proteins. (7) For l-tyrosine decarboxylase (*TdcA*, Enzyme Commission (EC) 4.1.1.25) and cytidine deaminase (*cCda*, EC 3.5.4.5), we found that genomic context is conserved between species and we also analyzed the genomic context. If the genomic context of a candidate gene was similar to that of a known drug-metabolizing gene, the candidate was considered as an ortholog of the known protein. Otherwise, it was considered to as a false positive prediction and excluded from further analysis (Supplementary Note 9 and Supplementary Fig. 10). As for (6), the tree was constructed again for only the orthologs of the known proteins. (8) For each tree, including only the orthologs of the known genes, we defined the monophyletic branches containing proteins derived from only one species. For each of such species-specific branches, we predicted subcellular localization (Supplementary Note 10) using the CELLO v.2.5 system (cello.life.nctu.edu.tw). (9) For cytoplasmic enzymes, drug transporters were predicted based on genomic context (Supplementary Note 11 and Supplementary Table 3b).

#### Tools

The PubSEED platform^53,56^ was used to annotate the subsystems. To search for BBHs for previously known proteins, a BLAST algorithm^63^ implemented in the PubSEED platform was used. Additionally, the PubSEED platform was used for analysis of the genomic context. To analyze the protein domain structure, we searched the Conserved Domains Database (CDD)^64^ using the following parameters: an e-value ≤ 0.01 and a maximum number of hits equal to 500. For the prediction of protein subcellular localization, the CELLO^65^ web tool was used. Alignments were performed using MUSCLE v.3.8.31 (ref. ^66^). For every multiple alignment, position quality scores were evaluated using Clustal X^67,68^. Thereafter, all positions with a score of zero were removed from the alignment and the modified alignment was used for construction of the phylogenetic trees. Phylogenetic trees were constructed using the maximum-likelihood method with the default parameters implemented in PhyML-3.0 (ref. ^69^). The obtained trees were midpoint-rooted and visualized using the interactive viewer Dendroscope, v.3.2.10, build 19 (ref.^70^).

### Literature and database searches

Biochemical and physiological characterization papers were retrieved by entering the names of AGORA2 species into PubMed (https://www.ncbi.nlm.nih.gov/pubmed/). Information on 132 carbon sources, 30 fermentation pathways, 64 growth factors, consumption of 73 metabolites and secretion of 51 metabolites was subsequently manually extracted on the species and/or genus level from 732 peer-reviewed papers and >8,000 pages of microbial reference textbooks^71^. Moreover, the traits of each reconstructed strain including taxonomy, morphology, metabolism and genome size were retrieved through database searches. The taxonomic classification of the strains was retrieved from NCBI Taxonomy (https://www.ncbi.nlm.nih.gov/taxonomy/). Information on morphology, habitat, body site, gram status, oxygen status, metabolism, motility and genome size was manually retrieved from the Integrated Microbial Genomes and Microbiomes^72^ database (https://img.jgi.doe.gov/) (Supplementary Table 1). All experimental data that were used to refine AGORA2 are available at https://github.com/opencobra/COBRA.papers/tree/master/2021_demeter/input.

### Generation of draft reconstructions

Draft reconstructions were generated through the KBase^24^ narrative interface. Genomes present in KBase were directly imported into the narrative. Otherwise, genomes in FASTA format were uploaded into the Staging Area and, subsequently, imported into the narrative through the ‘Batch Import Assembly From Staging Area’ (https://narrative.kbase.us/#catalog/apps/kb_uploadmethods/batch_import_assembly_from_staging) app. Genomes in FASTQ format were directly imported into the narrative through the ‘Import Paired-End Reads From Web’ (https://narrative.kbase.us/#catalog/apps/kb_uploadmethods/load_paired_end_reads_from_URL) app after retrieving the links to the corresponding files from https://www.ebi.ac.uk/ena/data/view/PRJEB23845 and https://www.ebi.ac.uk/ena/data/view/PRJEB10915. The imported assemblies were annotated using RAST subsystems^73^ through the ‘Annotate Multiple Assemblies’ (https://narrative.kbase.us/#appcatalog/app/RAST_SDK/annotate_contigsets) app. Draft metabolic reconstructions were generated through the ‘Create Multiple Metabolic Models’ (https://narrative.kbase.us/#appcatalog/app/fba_tools/build_multiple_metabolic_models) app and exported in SBML format through the ‘Bulk Download Modeling Objects’ (https://narrative.kbase.us/#appcatalog/app/fba_tools/bulk_download_modeling_objects) app.

### Semiautomated, data-driven refinement pipeline

We developed a semiautomated refinement pipeline, DEMETER^19^, which had been previously used to build AGORA^20^. Briefly, DEMETER was developed by testing gap-filling steps in few reconstructions and propagating identified solutions to many reconstructions. Curation against experimental data is performed in DEMETER by gap-filling the appropriate reconstructions with a complete pathway for each experimentally demonstrated function. Biomass production under aerobic and anaerobic conditions and on defined media as well as biosynthesis of cell wall components are also enabled through gap-filling solutions that had been previously determined in few reconstructions. Similarly, futile cycles are solved by identifying and correcting the affected reactions in few reconstructions and propagating these changes during the development of DEMETER. More details on DEMETER are provided in ref. ^19^. A detailed tutorial is available as part of the COBRA Toolbox^47^.

For the generation of AGORA2, we revised DEMETER substantially. Specifically, we (1) translated ~1,000 additional reactions and ~800 metabolites from KBase to VMH^23^ nomenclature; (2) introduced additional gap-filling reactions, where needed, to enable biomass production under anoxic conditions on a complex medium with thermodynamically consistent reaction directionalities; (3) removed futile cycles resulting in thermodynamically implausible ATP production by making the responsible reactions irreversible; (4) ensured through gap-filling and/or deletion of appropriate reactions that all reconstructions captured the collected experimental data; and (5) adjusted biomass objective functions to account for class-specific cell membrane and cell wall structures as well as introducing a periplasm compartment (Supplementary Note 3). As described previously^20^, all refinement and debugging solutions were manually determined for a subset of the reconstructions and subsequently propagated to many reconstructions, as appropriate. All newly included metabolites and reactions were formulated based on literature and/or database^23,28,74^ searches, while ensuring mass and charge balance through the reconstruction tool rBioNet^75^. Reactions identified through comparative genomics (Supplementary Table 3b,c) were added to up to 5,438 reconstructions. Non-gene-associated reactions, for which the respective gene could not be found through comparative genomics, were removed from the draft reconstructions if doing so did not abolish biomass production.

Curation efforts were verified via a test suite^19^. Specifically, it systematically tested whether each reconstruction (1) grew anaerobically on complex medium; (2) had correct reconstruction structure, that is, mass and charge balance, and correct syntax for gene–protein–reaction associations; (3) was thermodynamically feasible, for example, produced realistic amounts of ATP; and (4) captured known metabolic traits of the organism according to the collected experimental and comparative genomic data. Supplementary Table 2 summarizes all features that are tested by the test suite.

For consistency, the existing 818 AGORA1.03 reconstructions (v.25.02.2019, available at https://www.vmh.life/files/reconstructions/AGORA/1.03/AGORA-1.03.zip) also underwent refinement through DEMETER. The AGORA1.03 reconstruction of *Staphylococcus intermedius* ATCC 27335 was removed since it was a duplicate of the newly reconstructed strain *Streptococcus intermedius* ATCC 27335. The names of eight AGORA1.03 reconstructions were changed to correct strain determination and/or spelling (Supplementary Table 1).

DEMETER has been implemented in the COBRA Toolbox^47^ and was run in MATLAB (MathWorks) v.R2020b.

### Generation of quality control reports

The quality control reports and associated scores were determined for each AGORA2 reconstruction using the MetaboReport tool in the COBRA Toolbox^47^. The quality checks included are consistent with the Memote^42^ checks, as were the calculations of the scores. All 7,302 reports can be accessed via https://metaboreport.live.

### Formulation of the drug reactions

A literature search for microbial enzymes known to transform, degrade, activate, inactive or indirectly influence commonly prescribed drugs was performed, yielding 15 enzymes in total (Fig. 3a and Supplementary Table 5), which are encoded by 25 genes (Supplementary Table 3b). To enable comparative genomic analyses, only drug transformations that could be linked to specific protein-encoding genes were considered. As described above, enzyme-encoding genes were analyzed in their genomic context as outlined in ref. ^76^ using PubSEED subsystems^26,53^. Additional information on the presence of the analyzed genes was retrieved from refs. ^39,77,78^.

Literature and database searches were performed for the metabolic fate of commonly prescribed human-targeted drugs. The structures of 287 drug metabolites and drug degradation products were retrieved from 73 peer-reviewed papers, HMDB^79^, DrugBank^79^ and the Transformer database^80^. Reactions were formulated based on the collected experimentally determined drug structures, drug downstream product metabolite structures and reaction mechanisms. Both cytosolic and extracellular enzymatic reactions were formulated depending on the identified subcellular protein locations. Since at least six drugs undergoing glucuronidation in the human body have been shown to be substrates for the microbial ß-glucuronidase^81,82^ (Supplementary Table 6), it was assumed that all retrieved glucuronidated drug metabolites (118 in total) could serve as substrates. Additionally, ß-glucuronidase reactions were formulated for 33 glucuronidated drug metabolites from a previously reconstructed module of human drug metabolism^83^ and three glucuronidated hormones from Recon3D (ref. ^21^). New metabolites and reactions were assigned VMH IDs following standards in nomenclature used for COBRA reconstructions^9^, and formulated while ensuring mass and charge balance through the reconstruction tool rBioNet^75^. In total, for 98 drugs (Fig. 3b), 353 unique metabolites, 381 enzymatic reactions, 373 exchange reactions and 710 transport reactions (Supplementary Table 6a,b) were formulated.

### Atom–atom mapping

The COBRA Toolbox^47^ function ‘generateChemicalDatabase’ was used to generate atom–atom mappings. The process to obtain the atom–atom mappings for the AGORA2 reconstructions can be summarized as follows: (1) 1,894 out of 3,533 metabolic structures from the AGORA2 reconstructions were collected from the SMILES and InChIs associated with their metabolites and different chemical databases, such as VMH^23^, KEGG^74^, HMDB^79^, PubChem^84^ and ChEBI^85^ databases; the metabolic structures were standardized based on the InChI algorithm^86^ and can be found in the VMH database^23^; (2) the standardized metabolites and the reaction stoichiometry in the AGORA2 reconstructions were used to generate 5,583 out of 7,300 MDL RXN files; (3) 5,583 out of 7,300 AGORA2 reactions were atom mapped using the Reaction Decoder Tool algorithm^87^ for active transport reactions and a custom algorithm^47^ for passive transport reactions and coupled transport reactions. Atom–atom mappings can be found in the VMH database^23^ and are freely available at https://github.com/opencobra/ctf.

### Simulations

All simulations were performed in MATLAB (MathWorks) v.R2020b with IBM CPLEX (IBM) as the linear and quadratic programming solver. Computations were carried out on a tower with a 2.80-GHz processor and 64-GB RAM with 12 cores dedicated to parallelization. The simulations were carried out using functions implemented in the COBRA Toolbox^47^. Flux balance analysis (FBA)^34^ was used to simulate metabolic fluxes. All additional scripts for data generation, data analysis and data visualization are available at https://github.com/ThieleLab/CodeBase.

### Retrieval of reconstruction resources

Manually and semiautomatically curated reconstructions compared with AGORA2 were retrieved as follows: 72 fully manually curated reconstructions were downloaded from the BiGG database^28^ (http://bigg.ucsd.edu/). Reconstructions generated through gapseq^18^ (8,075 total) were downloaded from ftp://ftp.rz.uni-kiel.de/pub/medsystbio/models/EnzymaticDataTestModels.zip and exported in SBML format through the sybilSBML package in R using a custom script. MAGMA^17^ reconstructions (1,333 total) were downloaded from https://www.microbiomeatlas.org/data/MSP_GEM_models.zip. To enable comparability with AGORA2, exchange reactions in all retrieved reconstructions were translated to VMH^23^ nomenclature through custom MATLAB scripts. Moreover, an ATP demand reaction (VMH reaction ID: DM_atp_c_) was added if not already present and otherwise translated to VMH nomenclature.

### Generation of reconstructions through CarveMe

Protein fasta files corresponding to 7,279 AGORA2 strains were downloaded from either NCBI (https://www.ncbi.nlm.nih.gov/assembly) or ENA (https://www.ebi.ac.uk/ena) and subsequently used to run CarveMe. The remaining 23 AGORA2 strains were excluded as a corresponding protein FASTA file was not available. Reconstructions for 7,279 strains were generated with CarveMe^15^ v.1.5.1 on Python 3.7.13 (retrieved from https://www.python.org/downloads/release/python-3713) and relying on DIAMOND^88^ v.0.9.14.

### Generation of reconstructions through gapseq

Genome FASTA files retrieved as described above were used as the input for gapseq^18^. A total of 1,767 models were generated with gapseq 1.2, which was run in R^89^ v.4.1.2 on an Ubuntu 22.04 machine. The R interface of GLPK (package Rglpk) was used as the linear programming solver.

### Flux and stoichiometrically consistent reactions

The subset of flux and stoichiometrically consistent reactions, as defined in ref. ^29^, was retrieved through the ‘findFluxConsistentSubset’ and ‘findStoichConsistentSubset’ functions implemented in the COBRA Toolbox^47^. The fraction of stoichiometrically and flux consistent reactions, excluding exchange and demand reactions, was subsequently determined for each AGORA2 reconstruction and corresponding KBase draft reconstruction as well as for 5,587 reconstructions generated through CarveMe^15^, 8,075 reconstructions generated through gapseq^18^, 1,333 MAGMA^17^ reconstructions and 73 curated reconstructions from the BiGG database^28^. Briefly, the subset of stoichiometrically consistent reactions in a reconstruction includes all reactions that are mass and charge conserved, excluding exchange, demand and sink reactions, which are by definition mass and charge imbalanced^29^. The subset of flux consistent reactions consists of all reactions can carry flux under the defined set of constraints^29^.

### Validation against three independent experimental datasets

For an independent assessment of predictive potential of genome-scale reconstructions, independent (that is, not used for the reconstruction process) experimental data on metabolite uptake and secretion were retrieved from three sources^30,32,33^ and mapped onto the VMH^23^ nomenclature through custom MATLAB scripts. The experimental data included species-level positive and negative metabolite uptake and secretion data for 457 species (5,341 strains) and 269 metabolites in AGORA2 from the NJC19 resource^30^, and species-level positive metabolite uptake data from ref. ^32^ for 184 species (328 strains) and 85 metabolites in AGORA2. Moreover, strain-resolved positive and negative metabolite uptake and secretion data for 676 AGORA2 strains and 220 metabolites, and positive and negative enzyme activity data for 881 AGORA2 strains and 31 enzymes, were retrieved from the BacDive database^33^. The enzyme data were mapped to the respective reactions in each of the compared reconstruction resources’ namespaces. Positive data indicated that the metabolite uptake, secretion capability or enzyme activity had been demonstrated in a microorganism, while negative data indicated that the microorganism has been shown not to possess the capability. For each retrieved positive or negative data point, the capability of the respective model to take up or produce the corresponding metabolite was calculated using FBA on unlimited medium by either minimizing or maximizing the corresponding exchange reaction, respectively. For enzyme data, it was tested whether at least one reaction mapped to the respective enzyme was present in the model and could carry a nonzero flux. If the data point was positive and the corresponding model could also take up or secrete the metabolite or produce flux through the corresponding enzymatic reactions(s), this resulted in a true positive prediction, while a false negative prediction occurred when the microorganism was known to have this capability, but the corresponding model did not capture the trait. If the data point was negative and the corresponding model also could not take up or secrete the metabolite or did not produce flux through any reaction(s) mapped to the enzyme, this resulted in a true negative prediction, and otherwise the prediction was a false positive.

Prediction accuracies were calculated for the three experimental datasets. For an assessment of the predictive potential of AGORA2 compared with other reconstruction resources, the analysis was repeated for the strains in KBase draft reconstructions; CarveMe reconstructions; and BiGG, gapseq and MAGMA reconstructions that overlapped with the AGORA2 organisms with available data. To this end, the predictive value of all resources was tested via mixed effect logistic regressions with the in silico prediction as predictor and the in vivo behavior (binary) as response variable, while introducing the model as random effect variable accounting for the stochastic dependencies of predictions for different metabolites stemming from the same model. Moreover, the accuracy per model was calculated for all resources, and then compared with the AGORA2 accuracies via nonparametric sign rank tests. The list of all strains in the compared reconstruction resources that were tested against the three datasets is shown in Supplementary Table 4a. All scripts are available at https://github.com/ThieleLab/CodeBase.

### Validation of drug-metabolizing capacities against independent experimental data

A literature search was performed for in vitro experiments demonstrating the capabilities of human microbial strains to metabolize reconstructed drugs through the 15 annotated enzymes, resulting in 253 drug–microbe pairs (Supplementary Table 7). As this data contained both positive and negative data, true positive, true negative, false positive and false negative predictions could occur as described above. If no studies on the specific reconstructed drugs were found for the enzyme, studies on general activity of the enzyme were retrieved. If possible, the tested microorganisms were matched to AGORA2 models on the strain level, and otherwise pan-species models were used. Subsequently, the capabilities to metabolize the drugs through the respective enzymes for the 164 AGORA2 models with available data (Supplementary Table 7) were tested by computing whether the corresponding reaction could carry flux. Accuracy, sensitivity and specificity of predictions were calculated after determining the number of true positive, true negative, false positive and false negative predictions. *P* values were calculated by Fisher’s exact test and, for sensitivity analysis, by mixed effect logistic regression including the model as random effect variable, accounting for the stochastic dependency of predictions stemming from the same model.

### Drug yields

To determine each strain’s capability to metabolize drugs, all AGORA2 strains were constrained with a simulated Western diet^20^ and the flux through the exchange reactions corresponding to each drug was minimized using FBA, corresponding to maximal uptake rate of the drug. For all AGORA2 organisms capable to take up at least one drug, the yield of ATP, carbon and ammonia from 1 mmol of the drug per g_dry weight_ per h was evaluated as follows. Each reconstruction was constrained to only allow the uptake of water, phosphate and oxygen (VMH IDs: h2o, pi, o2). Demand reactions for ammonia as well as CO_2_ and pyruvate (as proxies for carbon sources) (VMH IDs: nh4, co2, pyr) were added, while a demand reaction for ATP (VMH ID: atp) already existed in each reconstruction. Next, the uptake of each drug metabolite (15 in total, one representative for each enzyme) was allowed one by one at an uptake rate of 1 mmol per g_dry weight_ per h. For each drug metabolite, the yields of ATP, ammonia, CO_2_ and pyruvate from each drug metabolite were computed using FBA by maximizing the flux through the respective demand reactions. As control, yields were also computed for 1 mmol per g_dry weight_ per h of glucose and without any metabolites added.

### Simulation of drug metabolism by individual gut microbiomes

Previously, metagenomic sequencing from fecal samples of a cohort of 616 Japanese patients with CRC and healthy controls had been performed^38^. Species-level abundances for this cohort, which have been determined with MetaPhIAn2 (ref. ^90^), were retrieved from https://www.nature.com/articles/s41591-019-0458-7#MOESM3. Unclassified taxa on the species level, eukaryotes and viruses were excluded. Of the remaining 517 species, 501 (97%) could be mapped onto the 1,738 AGORA2 species. Pan-species models for AGORA2 were created through the ‘createPanModels’ function. From the pan-species models, personalized microbiome models for each of the 616 samples were built through a computationally efficient pipeline^43^ with the species-level abundances as input data and parameterized as described elsewhere^10,60^. For each individual, we integrated all microbial models having a nonzero abundance in the sample into one personalized microbiome model. To contextualize the models with appropriate diet constraints, a simulated Average Japanese Diet described previously^41^ (Supplementary Table 12) was used. To predict the drug conversion potential of each microbiome, the fecal secretion reactions for 13 drug metabolism end products were optimized one by one using FBA^34^, while providing the respective precursor drug as well as oxygen at a de facto unlimited uptake rate of 1,000 mmol per g_dry weight_ per h.

### Shadow price analysis

To determine species in microbiome models that were of importance for the microbiome’s combined potential to metabolize a drug, a shadow price analysis was performed as described previously^60^. Briefly, shadow prices are a feature of every FBA solution (that is, the shadow price is the dual to the primal linear programming problem) that reflect the contribution of each metabolite in the model to the flux through the objective function^8^. A nonzero shadow price for a metabolite indicates that this metabolite has importance for the total flux capacity through the optimized objective function, that is, in our case, the secretion of a drug metabolic product. A shadow price of zero indicates that increasing the availability of this metabolite would not change the flux through the objective function. To determine the species that were bottlenecks for the conversion potential of the 13 drugs in each microbiome model, nonzero shadow prices for species biomass metabolites (‘species_biomass[c]’), which reflect the contribution of the species to the community biomass reaction, were retrieved.

### Statistical analysis

We analyzed statistically the net production capacity of 13 drug metabolites (Fig. 6a) among 252 healthy individuals and 364 patients with CRC. For each drug metabolite, we calculated the mean flux and the share of microbiomes with a flux greater than zero. Drug metabolites, which had in over 50% of the cases a zero flux, were dichotomized (can be produced versus cannot be produced) and, subsequently, analyzed via logistic regressions. Drug metabolites with over 50% nonzero entries were analyzed via linear regressions using heteroscedastic robust standard errors. First, we investigated potential effects of basic covariates (age, sex and BMI) via generalized linear regressions (logistic or linear) with the net production capacity being the response variable (dichotomized or metric). Age and BMI were introduced into the models as restricted cubic splines^91^ using four knots (the 5%-percentile, the 33%-percentile, the 66%-percentile and the 95%-percentile) resulting in three spline variables, each to test on potential nonlinear relationships. Significance was then determined by testing the three spline variables belonging to age (or BMI, respectively) simultaneously on zero via the Wald test^91^. While for age substantial nonlinearities were found, no indication for nonlinear BMI effects could be identified. The final models included, therefore, only the linear BMI term. Second, we tested for potential associations of net production capacities with case control status. This test was done via generalized linear regressions (logistic or linear) with the net production capacity being the response variable (dichotomized or metric), while adjusting for age (restricted cubic splines), sex (male/female) and BMI (linear). We corrected for multiple testing using the FDR, adjusting significance values for 13 tests per analyses stream. A test was considered nominal significant with *P* < 0.05 and FDR-corrected significant if FDR < 0.05. For sensitivity analysis, we recomputed the drug-metabolizing capabilities using an average European diet instead of a Japanese diet. Then, we calculated Pearson correlations for each drug metabolite between the secretion potentials under Japanese and an average European diet. All statistical analyses were performed with STATA 17/MP. All scripts are available at https://github.com/ThieleLab/CodeBase.

### Sign prediction of fecal metabolite–species associations using AGORA2-based community models

We utilized the publicly available metabolome dataset (*n* = 347) from ref. ^38^. To test whether AGORA2-based community modeling is capable of predicting the sign of statistical associations between species presence and fecal metabolite concentrations in the CRC sample, we calculated maximal net secretion for 52 metabolites with fecal metabolome data with more than 50% of the samples having concentrations above the limit of detection. Metabolite net secretion was computed using the mgPipe module in the Microbiome Modeling Toolbox^10,43^ while relying on computationally efficient flux variability analysis^92^. Then, we calculated for each species present in at least 10% of the microbiomes and at max 90% of the microbiomes the effect of species (binary predictor: species present versus species not present) on each fecal metabolite concentration in multivariable regressions, adjusting for age, sex, BMI and study group. We then filtered for all species metabolite associations with *P* < 0.05. Next, we calculated the effect of the species presence on the community net secretion of the corresponding metabolite in analogous regressions. Finally, we calculated for each metabolite the agreement in signs between the in vivo association statistics and the in silico association statistics. Significance was determined by Fisher’s exact test and FDR correction was applied, accounting for 52 tests. Note that the *P* values should be treated with care since the signs of the various association statistics may cluster due to the multivariate nature of both the metabolome and the microbiome data.

### Data visualization

The phylogenetic tree of AGORA2 organisms was constructed in PhyloT (https://phylot.biobyte.de/) and visualized in iTOL (https://itol.embl.de/)^93^. Violin plots were generated in BoxPlotR (http://shiny.chemgrid.org/boxplotr/). Clustering of taxa by reaction presence through *t*-distributed stochastic neighbor embedding (*t*-SNE)^52^ was performed using the *t*-SNE implementation in MATLAB with Euclidean distance, barneshut set as the algorithm and perplexity set to 30. Taxa with fewer representatives than 0.5% of all clustered strains were excluded from the *t*-SNE plots. Significance of differences in coordinates across taxonomic units was determined by Kruskal–Wallis tests. Circle plots were generated using the online implementation of Circos^94^. Figure 6 and Supplementary Fig. 9 were generated with the graphics functions of STATA 16/MP. All other data were visualized in MATLAB and R^89^.

### Reporting summary

Further information on research design is available in the Nature Portfolio Reporting Summary linked to this article.

## Online content

Any methods, additional references, Nature Portfolio reporting summaries, source data, extended data, supplementary information, acknowledgements, peer review information; details of author contributions and competing interests; and statements of data and code availability are available at 10.1038/s41587-022-01628-0.

## Supplementary information

Supplementary Information Supplementary Notes 1–11 and Figs. 1–10.

Reporting Summary

Supplementary Data 1 Supplementary Tables 1–12.

## Data Availability

The 7,302 AGORA2 reconstructions are freely available at https://www.vmh.life/ (https://www.vmh.life/files/reconstructions/AGORA2 for bulk download). Quality control reports for all reconstructions are available at https://metaboreport.live/.

## Code availability

Code and input data to reproduce the generation of the AGORA2 reconstructions and microbiome models as well as all simulations and analyses are available at https://github.com/ThieleLab/CodeBase.
