# Supplementary material for: Genome-scale metabolic reconstruction of 7,302 human microorganisms for personalized medicine
Source: Nat Biotechnol. 2023 Jan 19;41(9):1320–31. doi: 10.1038/s41587-022-01628-0 (PMC10497413; doi:10.1038/s41587-022-01628-0)
Supplement: Supplementary file 1 — Supplementary Notes 1–11 and Figs. 1–10. [file 41587_2022_1628_MOESM1_ESM.pdf]

nature biotechnology

Article https://doi.org/10.1038/s41587-022-01628-0

# Genome-scale metabolic reconstruction of 7,302 human microorganisms for personalized medicine

In the format provided by the authors and unedited

**Table of Contents**

**Supplemental Note 1:** AGORA 2.0 represents a substantial expansion in size and scope over AGORA 1.03
**Supplemental Note 2:** DEMETER results in high-quality reconstructions that capture known biochemical and physiological properties
**Supplemental Note 3:** Curation of biomass objective functions
**Supplemental Note 4:** Reconstruction features across taxa
**Supplemental Note 5:** Interspecies interactions and bottlenecks in microbial drug metabolism
**Supplemental Note 6:** Retrieval of genomes for human gut-associated strains
**Supplemental Note 7:** Curation of the subsystems, annotation of protein functional roles
**Supplemental Note 8:** Estimation of completeness of the analysed metabolic pathways
**Supplemental Note 9:** Manual refinement of the annotations for the drug-metabolising enzymes
**Supplemental Note 10:** Analysis of subcellular localisation of drug-metabolising enzymes
**Supplemental Note 11:** Prediction of drug transporting proteins
**Supplemental Figures S1-S10**
**Supplemental References**

**Supplemental Note 1: AGORA 2.0 represents a substantial expansion in size and scope over AGORA 1.03**

We extended the scope of AGORA2 beyond cultured and gut-associated strains found in Western microbiomes. While AGORA 1.03[1] mostly consisted of cultured species, AGORA2 includes 487 currently uncultured and/or uncharacterised strains (Figure 1a, Table S1). AGORA2 contains 737 bacterial isolates, including 105 novel species, from the Human Gastrointestinal Bacteria Culture Collection (HBC)[2]. AGORA2 accounts for body sites other than gut, such as skin and mouth (Table S1). While the selection of AGORA 1.03 strains was mainly based on species found in healthy young Western individuals[3], AGORA2 accounts for species detected in a cohort of elderly Parkinson's Disease patients and controls[4], a cohort of Japanese colorectal cancer patients and controls[5] as well as species isolated from Polynesian, Saudi, and Senegalese individuals[6]. The taxonomic extension of AGORA2 (Figure 2a, b) covers all 83 named microbes in the Broad Institute-OpenBiome Microbiome Library[7], all strains in the human gastrointestinal bacteria culture collection[2], 175 of 180 named species (97%) in 92,143 metagenome-assembled genomes from 11,850 human gut microbiomes[8], as well as 463 of the 560 named species (83%) in a resource of over 150,000 microbial genomes from 32 countries[9]. Finally, we expanded the coverage of AGORA beyond human-associated strains by reconstructing 127 mouse-associated strains (Figure 1a, Table S1), thus, enabling modelling of the mouse microbiome. Mouse models are an important tool in microbiome research, but the microbiome is distinct from human[10]. It should be noted that Bacilli and Gammaproteobacteria were overrepresented in the strain selection (Figure 1b) reflecting a likely sequencing bias for well-studied species and/or opportunistic pathogens.

**Supplemental Note 2: DEMETER results in high-quality reconstructions that capture known biochemical and physiological properties**

The AGORA2 reconstructions underwent continuous testing through a test suite[11] that ensured correct reconstruction structure, biochemical and thermodynamic consistency, as well as good agreement with experimental and genomic findings and known traits of the organism (Table S2). These efforts ensured that all curated reconstruction-derived condition-specific metabolic models could grow anaerobically on complex medium[11] and produced realistic amounts of ATP, which was not the case for models derived from most draft reconstructions (Figure 1c). We collected information on carbon sources, fermentation pathways, and growth requirements that served as input data for the pipeline. Using an iterative approach described in[11], the semi-automatically curated reconstructions were continuously tested against the input data. Discrepancies between experimental data and model predictions identified by the test suite were manually inspected and corrected (Methods). As a result, while the metabolic models derived from draft reconstructions showed low prediction accuracy, those derived from the curated AGORA2 reconstructions, as expected, agreed very well with the experimental data (Figure 1d). For instance, defined media had been reported for 74 AGORA2 strains[12] and all 74 reconstructions could grow on the respective media.

The metabolic reconstructions were further curated by mapping a published compendium of metabolite uptake and secretion data for ~570 human microbes[13] onto the AGORA2 strains. Corresponding exchange and transport reactions were added for each microbe reported to take up and/or secrete a metabolite. Thus, due to the extensive data-driven curation and refinement already performed, AGORA2 captured species-specific catabolic and biosynthetic pathways present in the human gut microbiome very well. Afterwards, the reconstructions were further improved by performing gap-filling to ensure the uptake and secretion of metabolites reported in[13], resulting in an agreement with metabolite uptake and secretion data of >99% for both (Figure 1d).

**Supplemental Note 3: Curation of biomass objective functions**

Gram-positive and -negative bacteria differ in their cell wall structure[14]. Gram-positive bacteria possess a thick layer of teichoic acid and an inner but no outer membrane, while gram-negative bacteria have an inner and an outer membrane separated by a large periplasmatic space with the outer membrane carrying lipopolysaccharides (LPS)[14]. As exceptions, the Deinococcus-Thermus phylum has on outer membrane, but does not have LPS[15], certain Firmicutes, such as *Acidaminobacter* and *Gracilibacter* sp. stain gram-negative, but have a gram-positive cell wall structure[14, 16], and the Chloroflexi phylum stains gram-negative, but has no LPS and no outer membrane[14]. Moreover, the Tenericutes phylum does not possess a cell wall[14] and archaea have ether lipids in their membranes instead of the bacterial ester lipids[14]. When inspecting the biomass objective functions (BOFs) in the draft reconstructions, it was found that 32% of reconstructions of gram-positive organisms had gram-negative components in their BOF or vice versa. Moreover, 35 Tenericutes and seven archaea draft reconstructions incorrectly had standard bacterial cell wall components in their BOFs. To correct this, a step adjusting the BOF according to taxon-specific cell wall structure was incorporated into DEMETER[11]. All BOFs were checked based on the respective organisms' gram status and corrected by removing incorrect metabolites and add teichoic acid or LPS, as appropriate. For gram-negative organisms with an outer membrane, a periplasmatic compartment was added. The exceptions listed above were taken into account. An archaeal BOF was formulated by retrieved ether lipid structures and the corresponding biosynthesis reactions from the reconstruction of *Methanosarcina barkeri*[17]. Gap-filling reactions enabling cell wall component production were also added to the corresponding reconstructions, if necessary.

**Supplemental Note 4: Reconstruction features across taxa**

The reaction content of the classes and families with the highest numbers of representatives in AGORA2 was visualised through t-distributed stochastic neighbour embedding (t-SNE)[18] (Methods, Figure 2a-d). Generally, reconstructions clustered by class and family indicating that related organisms were similar in reconstruction content (Figure 2a-b). However, multiple subclusters of microbial classes were observed, especially for the Bacilli and Gammaproteobacteria classes (Figure 2c-d). Clustering Bacilli and Gammaproteobacteria representatives separately revealed multiple subclusters in the *Enterococus*, *Staphylococcus*, and *Streptococcus* genera (Figure 2c) as well as in the *Escherichia* and *Salmonella* genera (Figure 2d). These clusters were already observed in the KBase draft reconstructions (Figures S2a-c) indicating that this was a result of the genome annotation.

The number of reactions, metabolites, and genes per reconstructions also varied by taxon (Figure 2e-g). The highest numbers of reactions, metabolites, and genes were found in the Proteobacteria phylum, likely as a result of many representatives of this phylum having large genomes (Table S1) and a generalist type of metabolism[14]. The smallest reconstructions were found in the Tenericutes phylum, which consisted of organisms without a cell wall[14]. Growth rates on a previously defined Western diet[1] also correlated with reconstruction size and were in a biologically realistic range (Figure 2h).

**Supplemental Note 5: Interspecies interactions and bottlenecks in microbial drug metabolism**

For some drug metabolic products, only a subset of microbiome samples correlated with the abundances of the enzymes directly producing them (Figure 5b). We hypothesised that this was due to multiple species being involved in the metabolism of these drugs. An interspecies interaction in the metabolism of the Parkinson's Disease drug levodopa has been demonstrated

previously[19]. To identify species and enzymes that were bottlenecks for drug metabolism, we performed a shadow price analysis as described previously[20] (Methods). Non-zero shadow prices of biomass metabolites indicated that these species were flux bottlenecks for the drug metabolic product (Methods, Figure S6, Table S10). For instance, for the conversion of the anticancer prodrug 5-fluorocytosine to the active drug 5-fluorouracil via cytosine deaminase, and the detoxified end product 5,6-dehydro-5-fluorouracil via dihydrouracil dehydrogenase, either the first or the second step were flux bottlenecks (Figure S6a). Examples are *Anaerotruncus colihominis* and *Hungatella hathewayi*, which carry only cytosine deaminase, and *Escherichia coli*, which has both cytosine deaminase and dihydrouracil dehydrogenase (Figure S6a). For the previously described case of levodopa metabolism[19], two variations of flux limitations could be distinguished. If production of the end product m-tyramine correlated directly with dopamine dehydroxylase abundance, the abundance of *Eggerthella lenta*, the only species carrying this enzyme was flux-limiting (Figure S6b). In contrast, for microbiomes outside the correlation curve, the abundance of species carrying tyrosine decarboxylase, which produces dopamine from levodopa (e.g., *Enterococcus* sp.), was flux limiting (Figure S6b). A species-species interaction in levodopa metabolism involving *Enteroccocus* sp. and *E. lenta* had been previously reported[19]. Since this microbial pathway lowers the bioavailability of the active drug levodopa[19], identifying the species that serve as bottlenecks for levodopa conversion is an important consideration for Parkinson’s Disease treatment strategies.

**Supplemental Note 6: Retrieval of genomes for human gut-associated strains**
An initial set of genomes for a comparative genome analysis included 632 of 773 genomes from the AGORA genome set[1]. To extend this set, we retrieved genomes for all the strains for microbial species associated with human gut[21], and the genomes for 4,881 of such strains were available at the PubSEED resource. To check the quality of genome sequencing and assembly, we analysed the distribution of 31 genetic marker genes that are these proteins because they are nearly universally distributed in Bacteria and exist as single copy genes within each genome[22]. In agreement with the distribution of the genetic marker genes, this gene list was modified. Thus, the genes *dnaG*, *infC*, and *pgk* were excluded because these genes often exist as multiple non-identical copies within certain genome, whereas the gene *pyrG* was excluded because its absence in multiple analysed genomes. Based on the distribution of the other genetic marker genes, 43 genomes were excluded from the genome set as they lacked multiple genetic marker genes and/or had the presence of multiple identical copies of the gene in a single genome. Thus, genome set used for the comparative genomics manual refinement included 4,848 genomes. For an analysis of drug metabolism, the genome set was further extended by adding 643 genomes for other gut associated strains that had been retrieved through literature searches (Supplemental Note 1) and were available in the PubSEED database. Thus, for drug metabolism, genome set was extended up to 5,438 microbial genomes.

**Supplemental Note 7: Curation of the subsystems, annotation of protein functional roles**
All the functional roles corresponded to a single catalysed reaction, or a set of catalysed reactions (see below), were grouped into subsets, if possibly. For example, NADH- and NADPH- specific forms of FMN-dependent azoreductases (EC 1.7.1.6 and EC 1.7.-.-, respectively) were attributed to different subsets. Two types of the subsets were created, (1) including all subunits for certain enzyme or transporter, such as catalytic and electron-transfer subunits of digoxin reductase, and (2) including alternative names for the same enzymatic activity, such as b-galactosidases (EC 3.2.1.23) of the families GH2, GH35, and GH42. The single protein can belong to more than one subset, for example, NAD(P)H-specific form of azoreductase was attributed to subsets for both NADH- and NADPH-specific forms, however, this protein is not homologous to any of two previous.

To include all the alternative names for the same enzyme or transporter, chromosomal gene clusters were analysed. This was done for all genes that were absent in a certain genome but were present genomes of organisms belonging to the same species or genus. The following procedure was applied: (1) Genome context of the analysed gene was compared and genes, clustered with the analysed gene in multiple genomes of the related organisms were determined. (2) Orthologs of the clustered genes were searched in the analysed genome, and, if they were found, (3) their genomic context in the analysed genome was studied to find a gene that (i) have a genome context similar to that was observed in the genomes of the related organisms for the analysed gene and (ii) have a name synonymous to the name of the analysed gene or other genes in the subset including the analysed gene. (4) If such candidate was found, a new functional role was included into the subsystem and included into the corresponding subset.

**Supplemental Note 8: Estimation of completeness of the analysed metabolic pathways**

Most of the curated subsystems correspond to biosynthesis of certain metabolite(s). All the biosynthetic pathways for each of potentially synthesised metabolite were collected and analysed. More than one pathway may be possible for a single metabolite, as well the same gene can be included into different pathways for the synthesis of different metabolites.

Based on the presence of enzymatic genes, all the pathways were classified to the following categories. (1) Complete pathways included genes for all the enzymes of the pathway. (2) Gapped pathways were defined as that have no genes for no more than two reactions and length of each gap in the pathway does not exceed one reaction. (3) For incomplete pathways genes for more than two reactions were absent or extension of a gap exceeded two or more reactions. (4) If no gene of a pathway was present in the genome, the pathway was defined as absent. For the incomplete pathways, no reactions corresponding to the present genes were included into metabolic reconstructions. For the gapped pathways, a gap-filing procedure was applied, whereas for the complete pathways, reactions for all the genes were included into the reconstructions. The metabolite was considered as being synthesised by a microorganism if at least one complete or gapped biosynthetic pathway was predicted. This classification was applied to all analysed pathways except the (1) drug and bile acids transformations, which have been shown to rely on microbial collaboration to complete a pathway[19]; (2) respiration because all the curated pathways consist of only one reaction, and (3) central carbon catabolism because these pathways are tightly connected to the biosynthetic pathways and may be defined as incomplete whereas not generating blocked reactions[1].

**Supplemental Note 9: Manual refinement of the annotations for the drug-metabolising enzymes**

For a refinement of functional annotations for the drug-metabolising enzymes the following procedure was applied. (1) Phylogenetic tree was constructed for protein sequences of all the found BBHs. (2) Experimentally confirmed drug-metabolising enzymes were mapped onto the tree. (3) Monophyletic branches containing the experimentally confirmed enzymes were defined. (4) Branches lacking these enzymes were considered as false-positive predictions and were excluded from the analysis (Figure S10). Additionally, it was found that two of the drug-metabolising enzymes have a conserved genomic context. Thus, a gene for L-tyrosine decarboxylase (TdcA, EC 4.1.1.25) is clustered together with a gene for tyrosyl-tRNA synthetase (EC 6.1.1.1), whereas a gene for cytidine deaminase (cCda, EC 3.5.4.5) is clustered together with genes for pyrimidine-nucleoside phosphorylase (EC 2.4.2.2) and deoxyribose-phosphate aldolase (EC 4.1.2.4). Such a genomic context was used to distinguish between

genes for the drug-metabolising enzymes (clustering is conserved) and their paralogs (no clustering).

**Supplemental Note 10: Analysis of subcellular localisation of drug-metabolising enzymes**
For all the drug-metabolising enzymes the following procedure was applied. (1) For all the enzymes homologous to each other, maximal-likelihood phylogenetic tree was constructed; for example, for β-galactosidases, three trees were constructed for the GH2, GH35, and GH42 families, respectively. (2) For every tree, species-specific monophyletic branches were defined. (3) Subcellular localisation was predicted with CELLO[23, 24] web tool for one randomly selected protein for every species-specific monophyletic branch and then extrapolated to the whole species-specific monophyletic branch.

**Supplemental Note 11: Prediction of drug transporting proteins**
Cytoplasmic drug metabolising enzymes require the presence of transporters delivering corresponding drugs into the cytoplasm. To predict these transporters, we analysed genomic context for the predicted cytoplasmic enzymes. Candidate drug transporters should satisfy the following criteria: (1) Genes for these transporters should be chromosomally co-localised with the genes for the cytoplasmic enzymes. (2) This co-location should be evolutionary conserved, i.e., should be observed in more than one species. (3) Genes for candidate drug transporters should demonstrate sequence similarity with known domains specific for transport proteins, which was checked by search on CDD database[25] using the following cut off parameters: an e-value ≤0.01 and a maximum number of hits equal to 500.

## Supplemental Figures

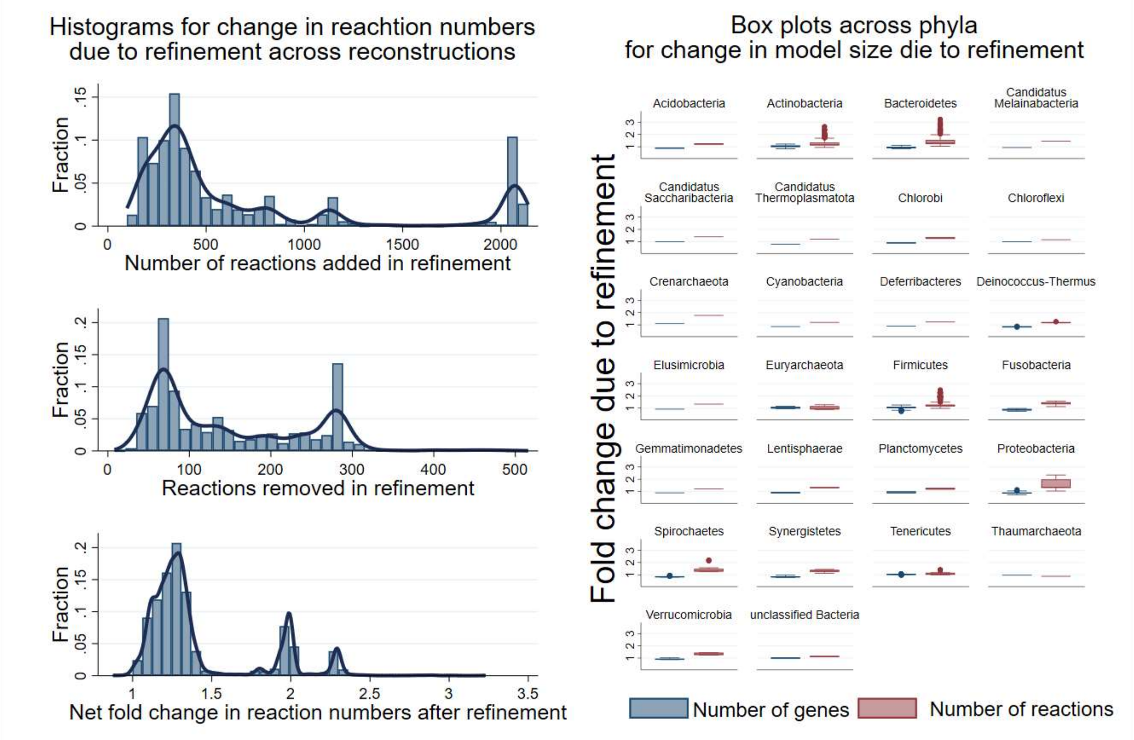


**Figure S1:** Change in model size due to refinement of reconstruction for the 5,438 reconstructions, for which comparative genomics were performed. **Left Panel**: Histograms for change in reaction numbers across reconstructions. **Right panel**: Box plots across phyla for fold change in gene and reaction numbers due to reconstruction. High numbers of added reactions were result of adding drug reactions to reconstructions with drug-metabolising potential. Boxes of box plots were defined by 25th percentile, the median, and the 75th percentile. Whiskers cover 1.5 times the interquartile range.

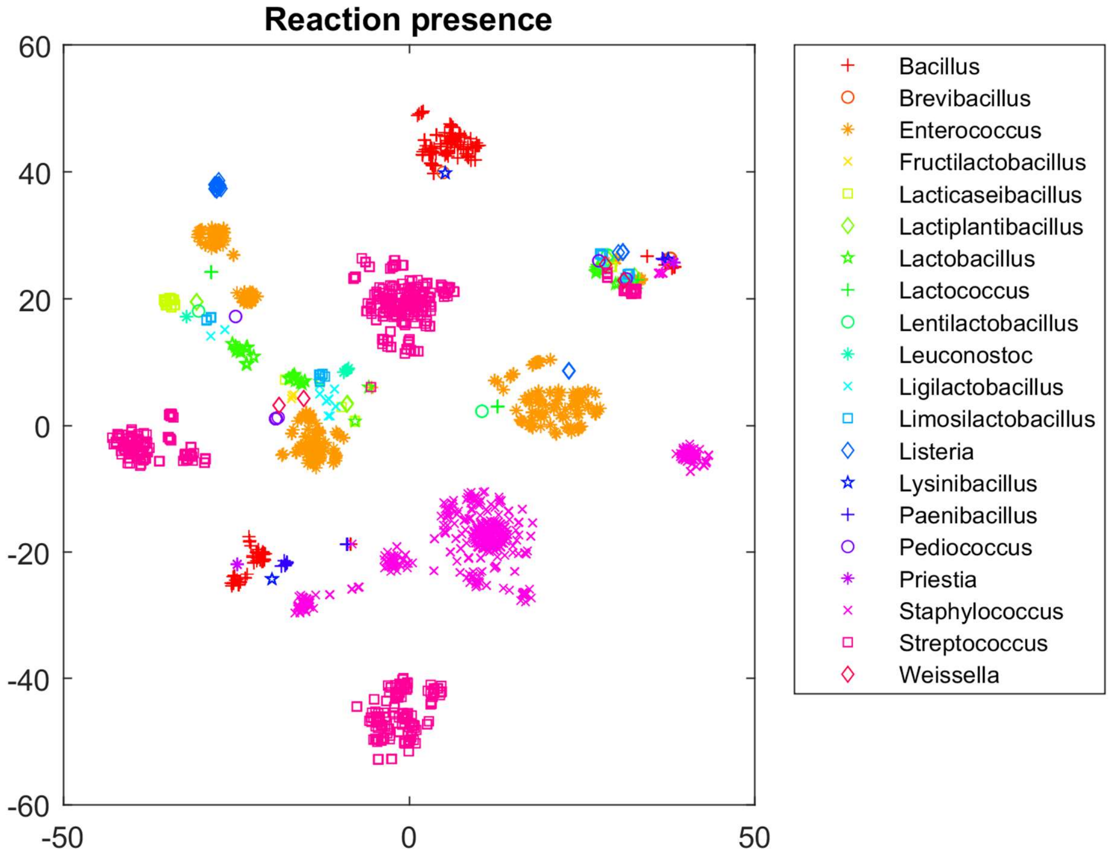


**Figure S2a)**: Clustering through t-distributed stochastic neighbour embedding (t-SNE) of reaction presence across all pathways per reconstruction for the draft reconstructions retrieved from KBase. Shown are the members of the Bacilli class by genus.

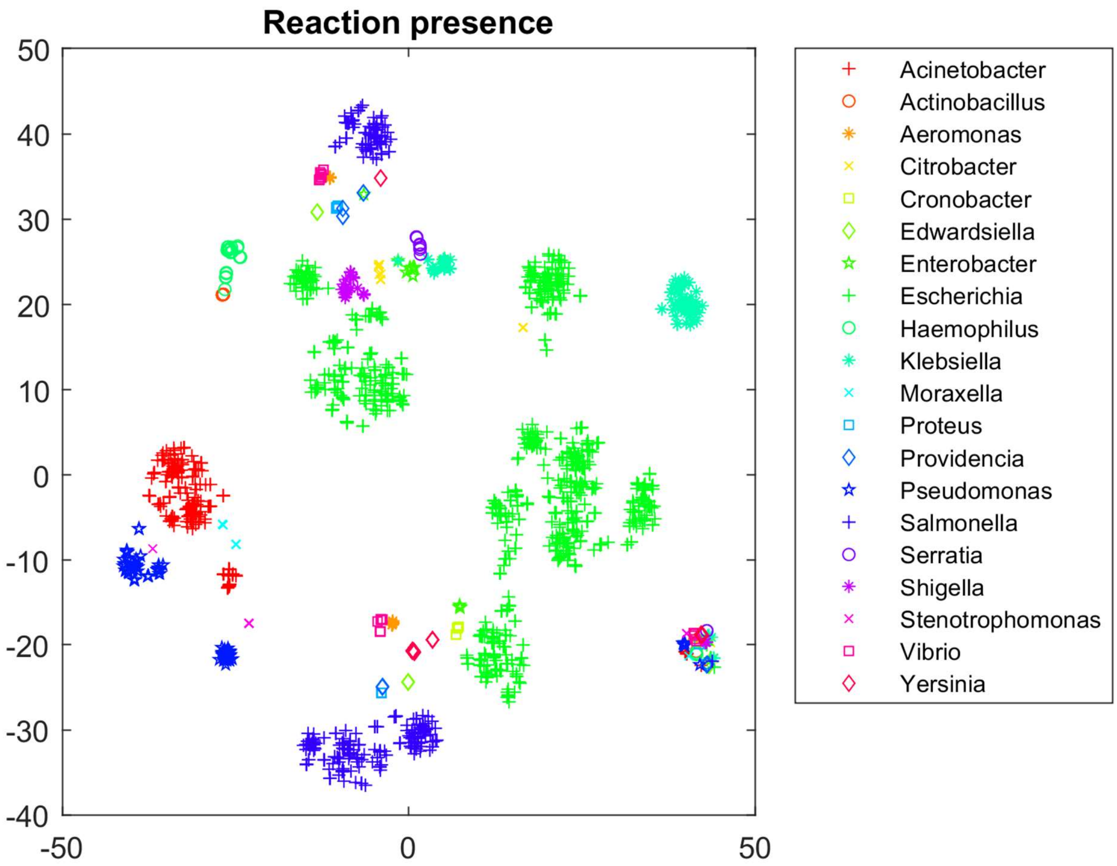


**Figure S2b)**: Clustering through t-distributed stochastic neighbour embedding (t-SNE) of reaction presence across all pathways per reconstruction for the draft reconstructions retrieved from KBase. Shown are the members of the Gammaproteobacteria class by genus.

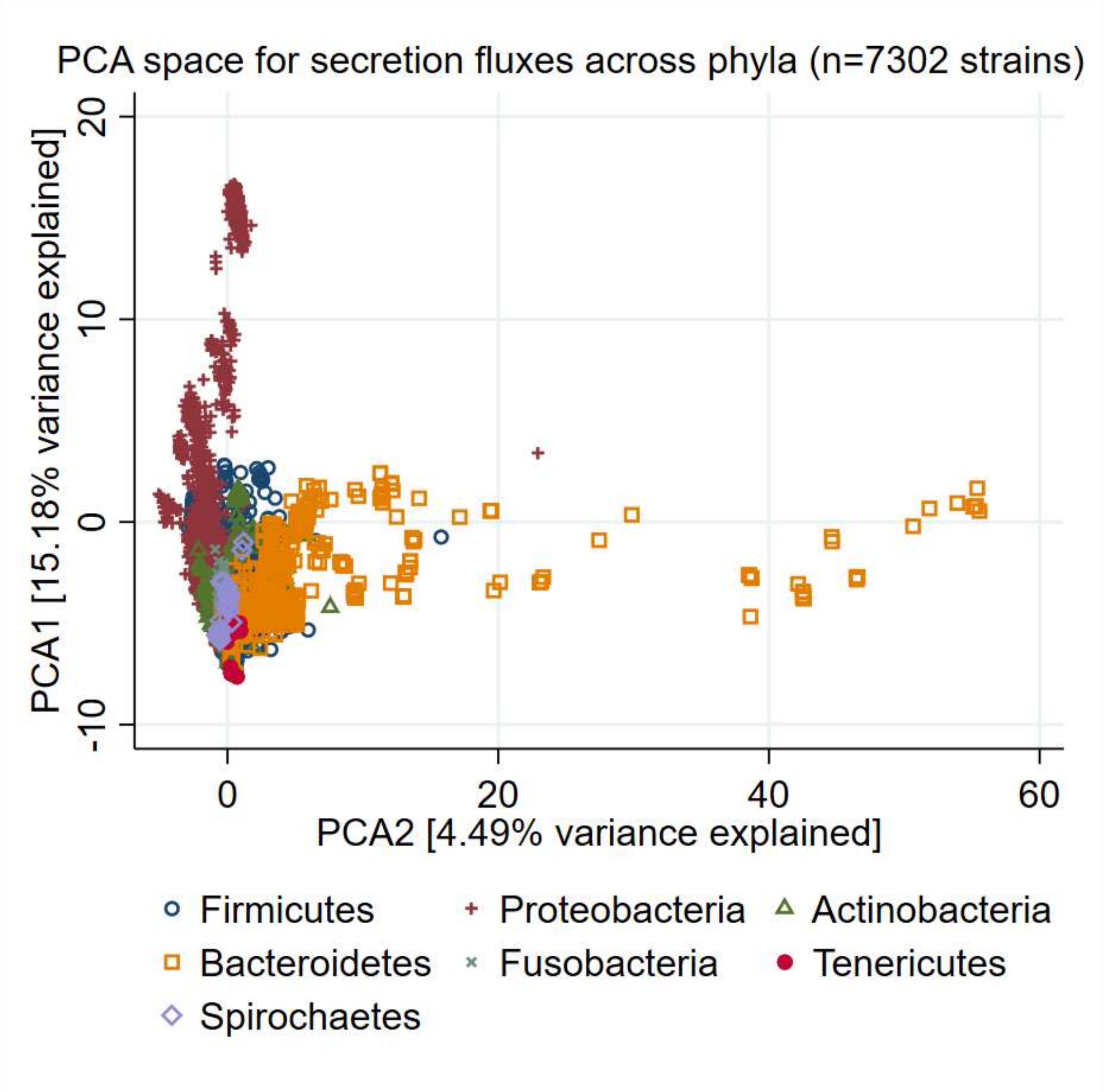


**Figure S3a**: Principles Component Analysis (PCA) space (first two dimensions) of the uptake fluxes under a European diet for 7,302 strains. Displayed are the strains belonging to the seven biggest phyla in AGORA2 (Firmicutes, Proteobacteria, Actinobacteria, Bacteroidetes, Fusobacteria, Tenericutes, Spirochaetes).

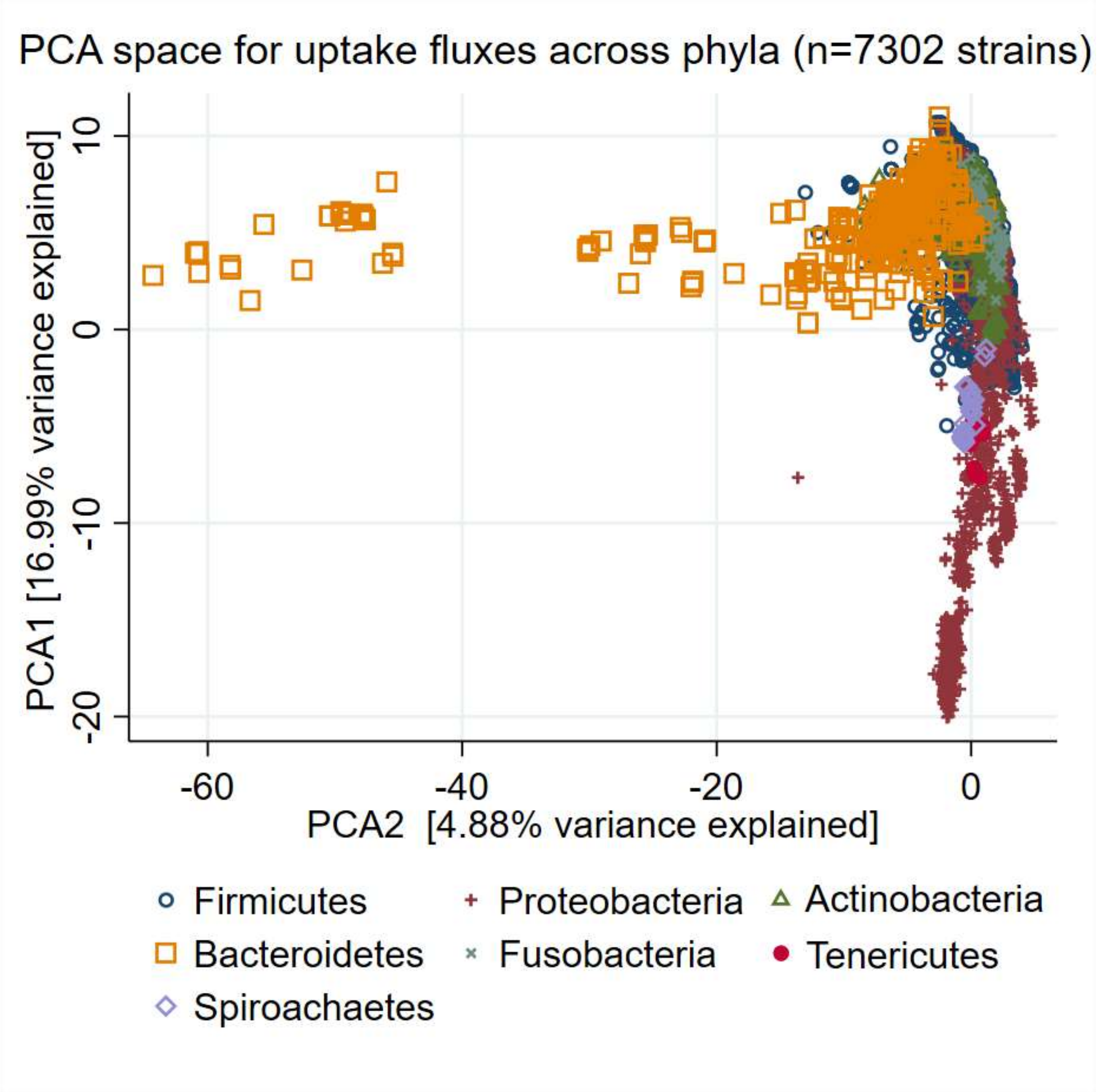


**Figure S3b**: Principles Component Analysis (PCA) space (first two dimensions) of the secretion fluxes under a European diet for 7,302 strains. Displayed are the strains belonging to the seven largest phyla in AGORA2 (Firmicutes, Proteobacteria, Actinobacteria, Bacteroidetes, Fusobacteria, Tenericutes, Spirochaetes).

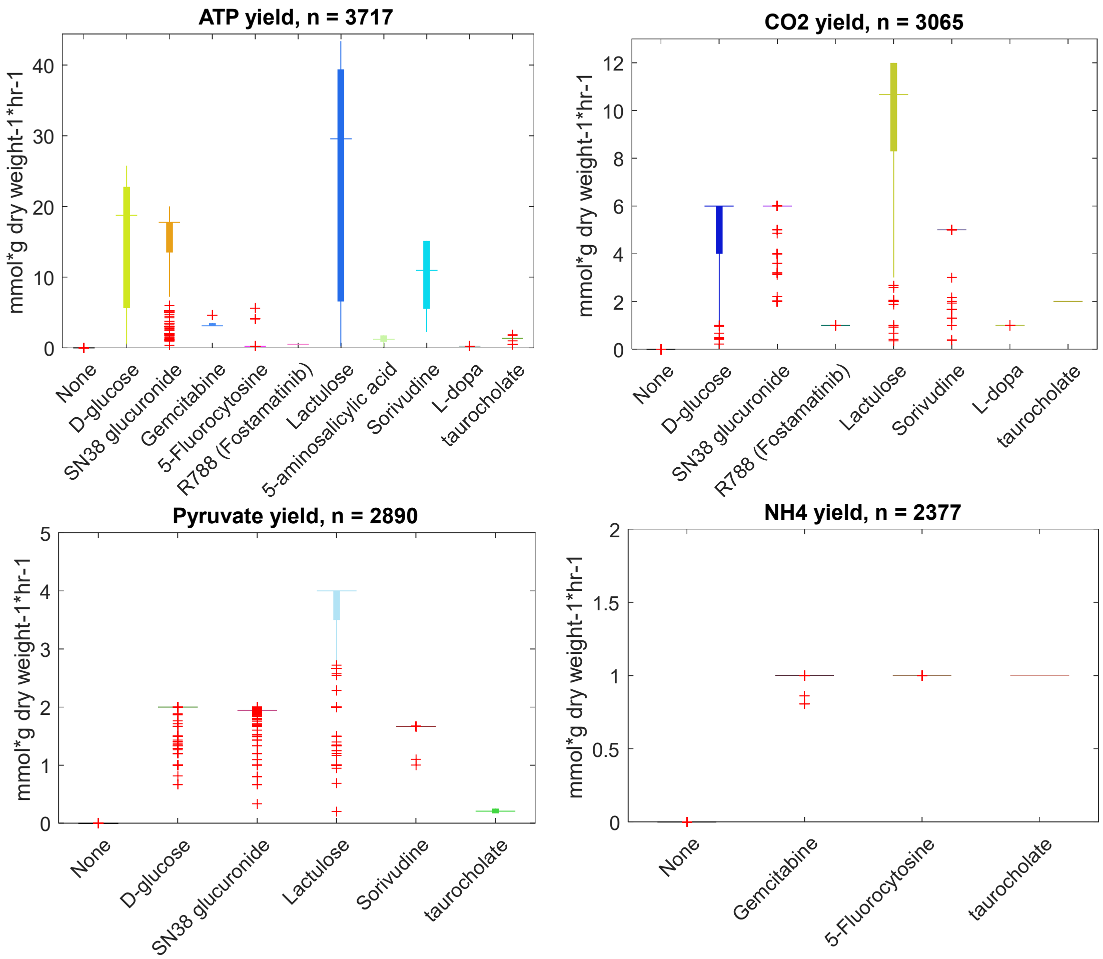


**Figure S4**: Yields from 1 mmol/$g_{dry\ weight}$/hr of drugs that can serve as sources for ATP, $CO_2$, pyruvate, and $NH_4$ production. Shown are all microbes that could use at least one drug to produce the respective source. Flux with glucose and with no compound added are shown as controls. One example drug per enzyme was tested.

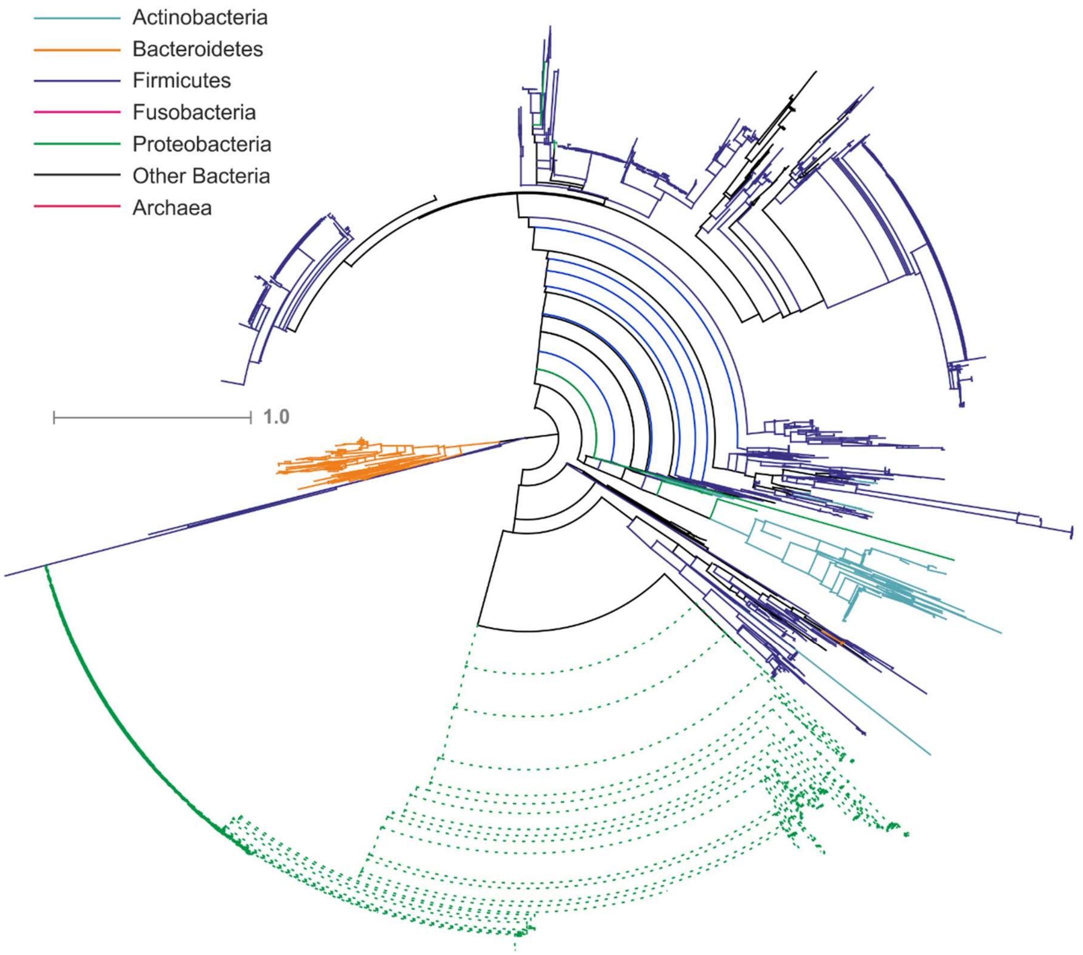


**Figure S5a:** Maximal-likelihood phylogenetic tree for cytidine deaminase (cCda, eCda, EC: 3.5.4.5) proteins in the analysed genomes. Taxonomy is shown by branch colour; solid lines, cytoplasmic proteins; dotted lines, extracellular / periplasmic proteins.

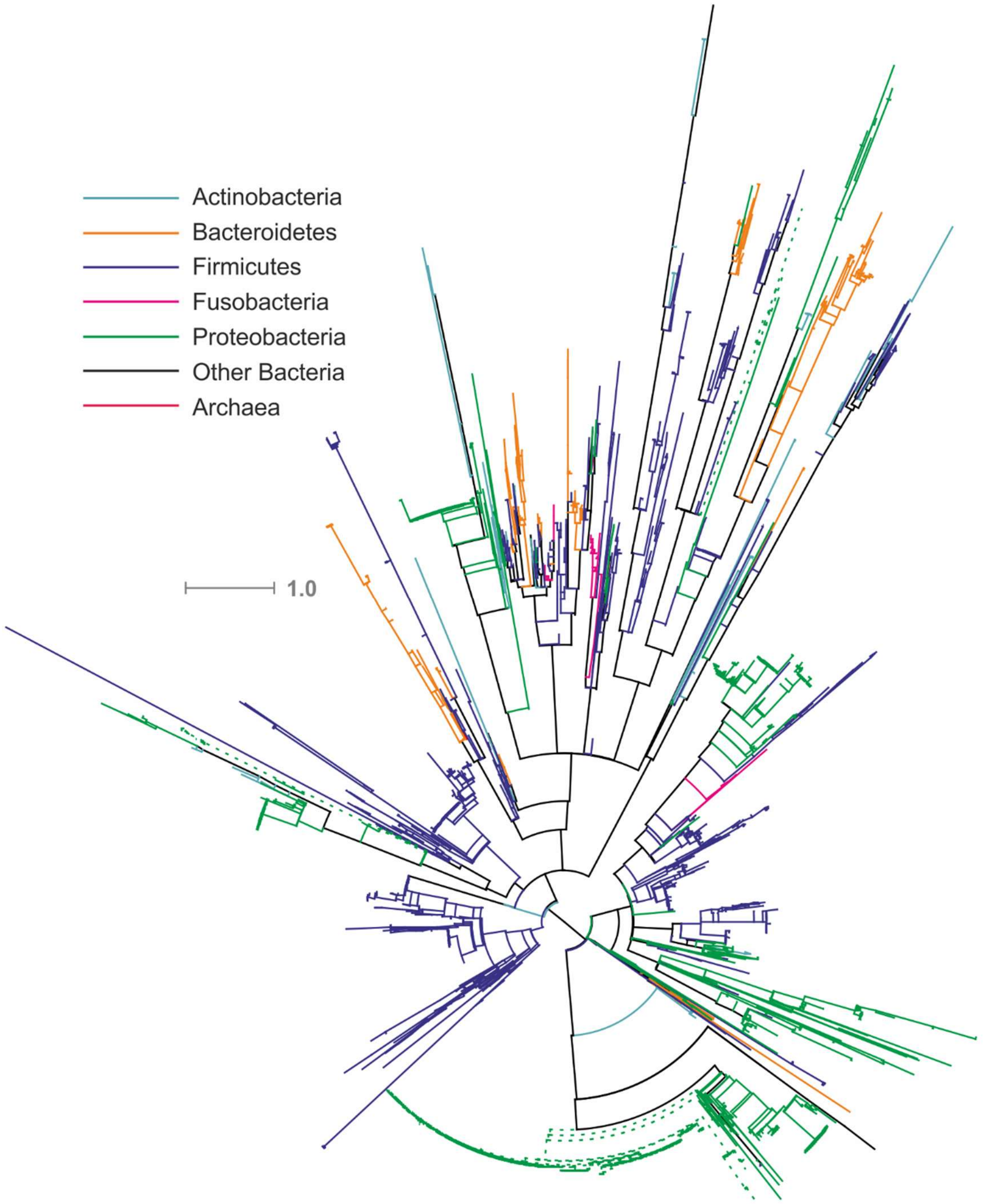


**Figure S5b:** Maximal-likelihood phylogenetic tree for nitroreductase (cNit, eNit, EC: 1.-.-.-) proteins in the analysed genomes. Taxonomy is shown by branch colour; solid lines, cytoplasmic proteins; dotted lines, extracellular / periplasmic proteins.

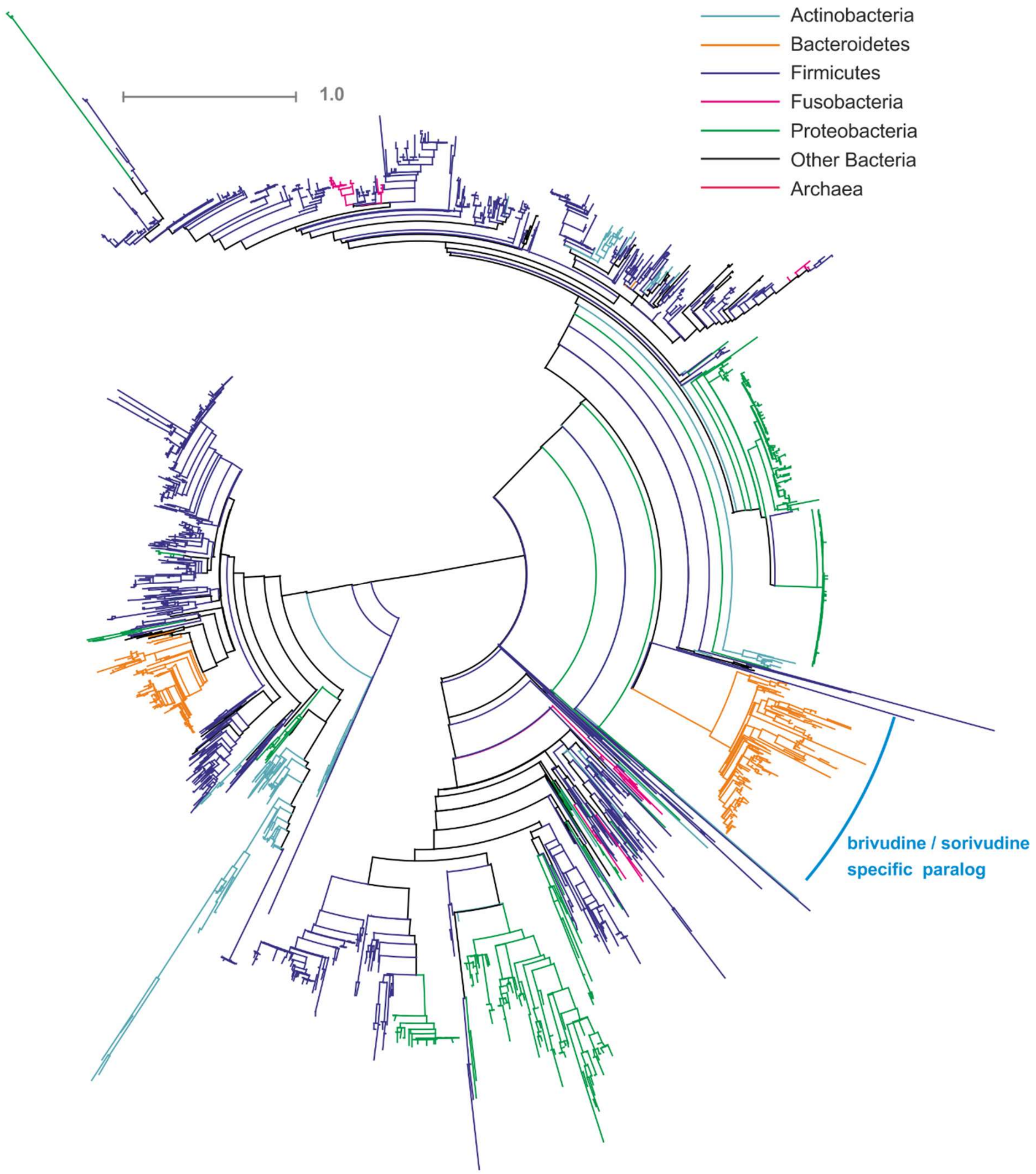


**Figure S5c:** Maximal-likelihood phylogenetic tree for pyrimidine-nucleoside phosphorylase (cBRV, EC: 2.4.2.2) proteins in the analysed genomes. Taxonomy is shown by branch colour.

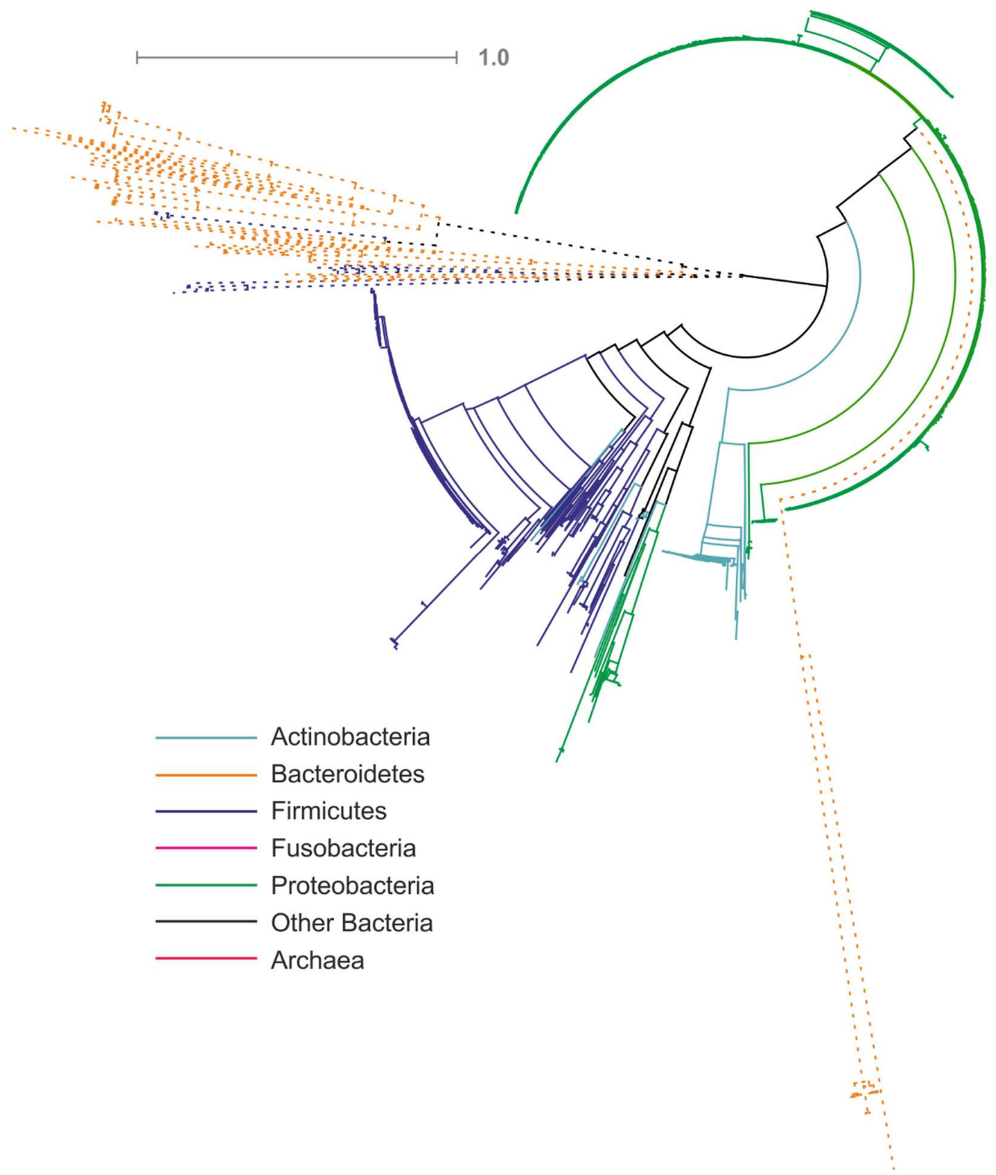


**Figure S5d:** Maximal-likelihood phylogenetic tree for β-glucuronidase (cUidA, eUidA, EC: 3.2.1.31) proteins in the analysed genomes. Taxonomy is shown by branch colour; solid lines, cytoplasmic proteins; dotted lines, extracellular / periplasmic proteins.

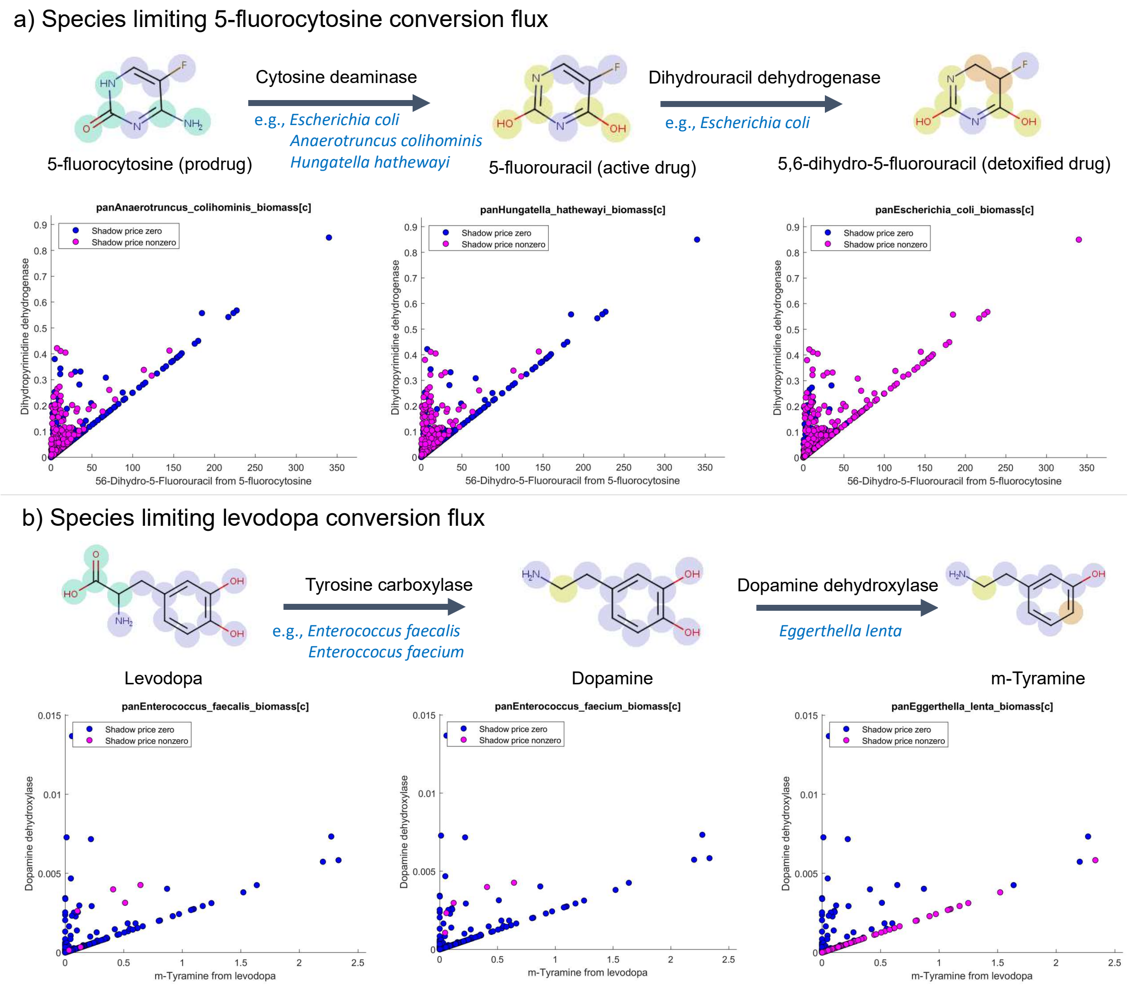


**Figure S6: Bottlenecks limiting drug-metabolising capacity in 616 microbiomes.** Non-zero shadow prices indicate that increasing the abundance of this species would increase the secretion flux of the end product of the shown enzymatic reaction in this microbiome. A shadow price of zero shows that increasing the abundance of the species would not affect secretion of the end product. a) Pathway of 5-fluorocytosine deamination to 5-fluorouracil and subsequent reduction to 5,6-dihydro-5-fluorouracil. b) Pathway of levodopa decarboxylation to dopamine and dopamine dehydroxylation to m-tyramine. In each panel, the x axis shows net secretion flux of the drug metabolite per microbiome in mmol/$g_{dry\ weight}$/day and the y axis shows the relative reaction abundance per microbiome.

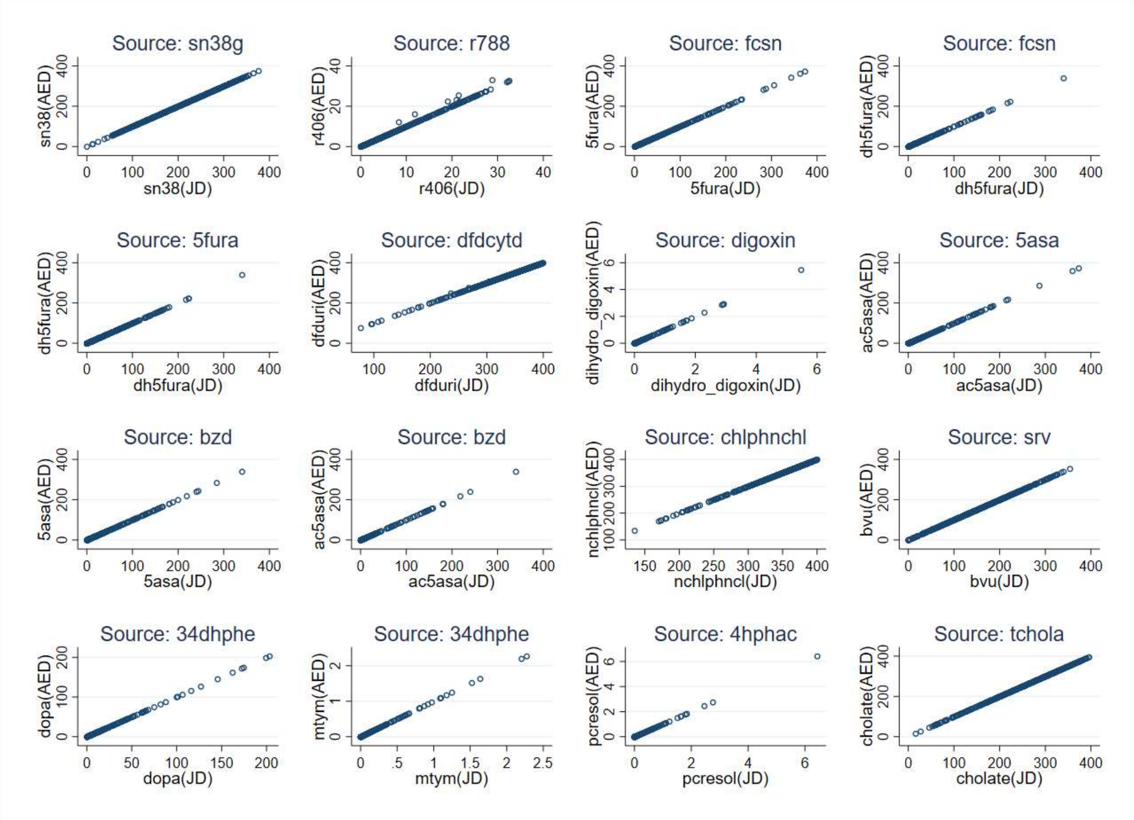


**Figure S7:** Scatter plots of drug-metabolising potentials under a European average diet (AED) against the metabolising potential under a Japanese diet (JP). sn38g=glucuronated irinotecan, sn38=deglucuronated irinotecan, 5fura=5-fluorouracil, fcsn=5-fluorocytosine, dh5fura=5,6.dihydro-5-fluorouracil, dihydro_digoxin=Dihydrodigoxin, ac5asa=N-acetyl-5-aminosalicylic acid, 5asa=5-aminosalicylic acid, dfduri=2',2'-Difuorodeoyuridine, ac5asa_bzd=N-acetyl-5-aminosalicylic acid from balsalizide, chlphncl=chloramphenicol, nchlphncl=Nitrosochoramphenicol, bvu=(E)-5-(2-Bromovinyl)Uracil, 34dhphe=levodopa, dopa=Dopamine, mtym=m-tyramine, 4hphac=p-Hydroxyphenylacetic acid, tchola=taurocholate, cholate=cholic acid.

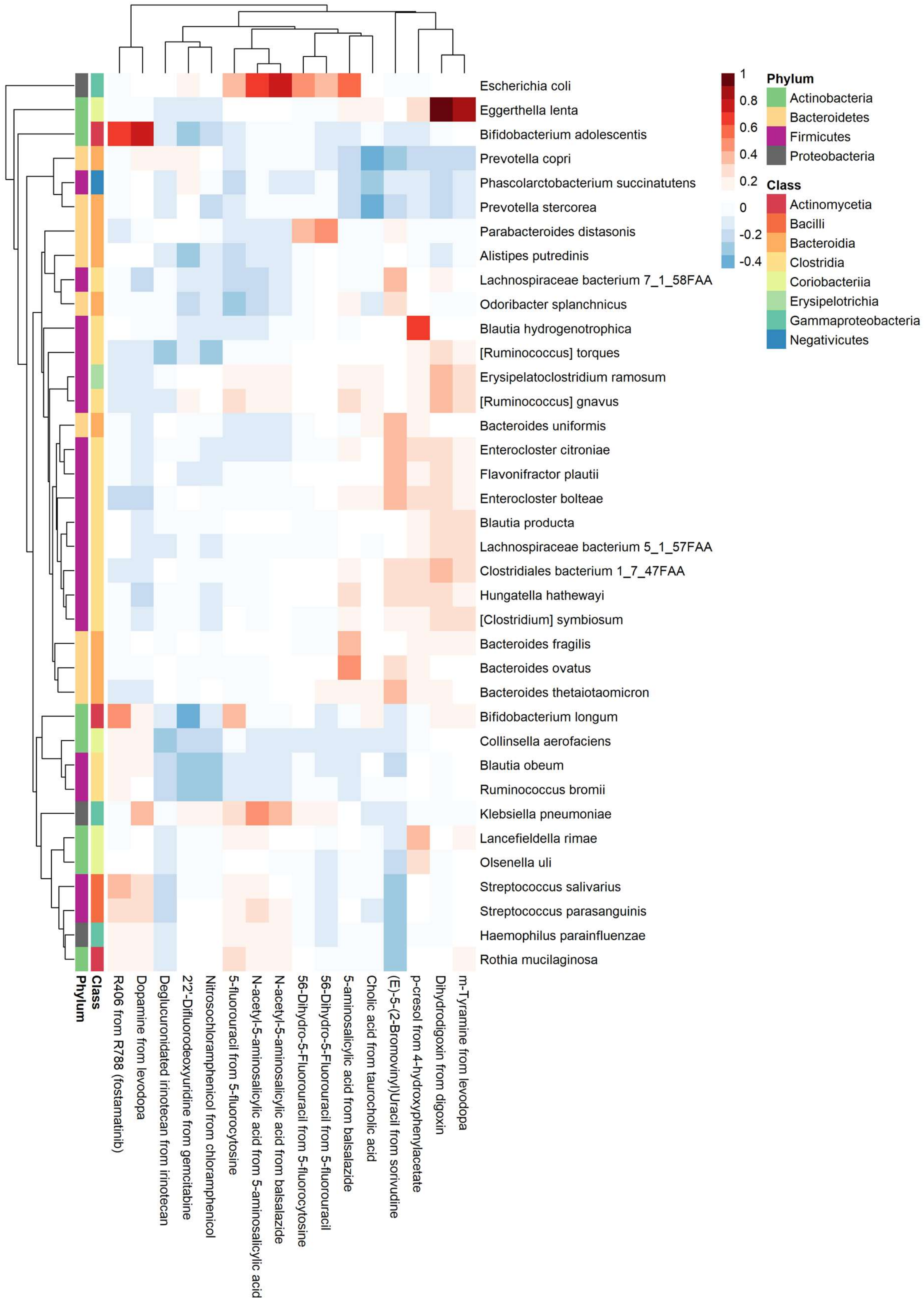


**Figure S8**: Spearman correlations between species abundances and drug conversion potential (mmol/person/day) in 616 microbiomes of Japanese colorectal cancer patients and controls.

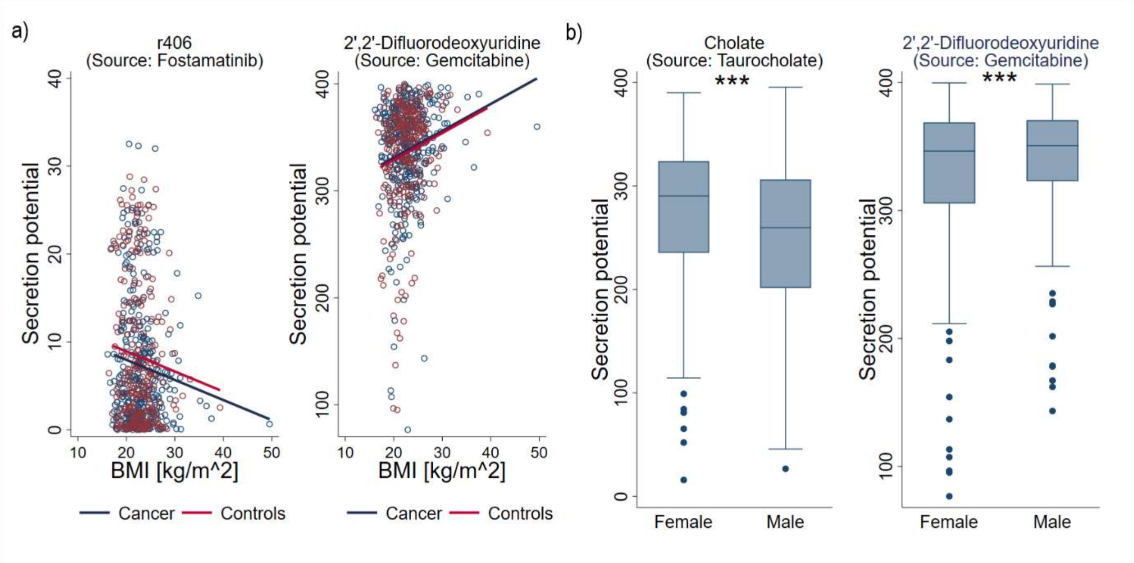


**Figure S9**: Descriptive statistics for the modelled drug metabolites. a) Scatter plot (red: controls; blue cancer) of 2',2'-difluorodeoxyuridine (microbial metabolite of 5-fluorocytosine) and r406 (metabolite of fostamatinib) in dependence of BMI with linear regression lines for cases and controls. The slope of BMI was significant (2',2'-difluorodeoxyuridine: b=2.11, 95%-CI=(1.12;3.09), p=1.06e-05, FDR<0.05; r406:b=-0.22, 95%-CI=(:-0.37;-0.06). p=3.09e-03, FDR<0.05) adjusted for sex and age (restricted cubic splines), but no significant differences could be found between CRC cases and controls (difluorodeoxyuridine: p=0.77; r406: p=0.27). b) Box plots of 2',2'-difluorodeoxyuridine (metabolite of gemcitabine), cholate (metabolite of taurocholate) on sex. P-values were derived from linear regressions adjusted for age (restricted cubic splines). All effects were significant after correction for multiple testing (2',2'-difluorodeoxyuridine : b=13.62, 95%-CI=(5.42;21.83), p-value: 1.17e-03, FDR<0.05; cholate: b=-25.10, 95%-CI:(-39.90;-13.29), p=3.40e-05, FDR<0.05). Boxes of box plots are defined by the 25th percentile, the median, and the 75$^{th}$ percentile. Whiskers cover 1.5 times of the interquartile range. All p-values are reported two-sided.

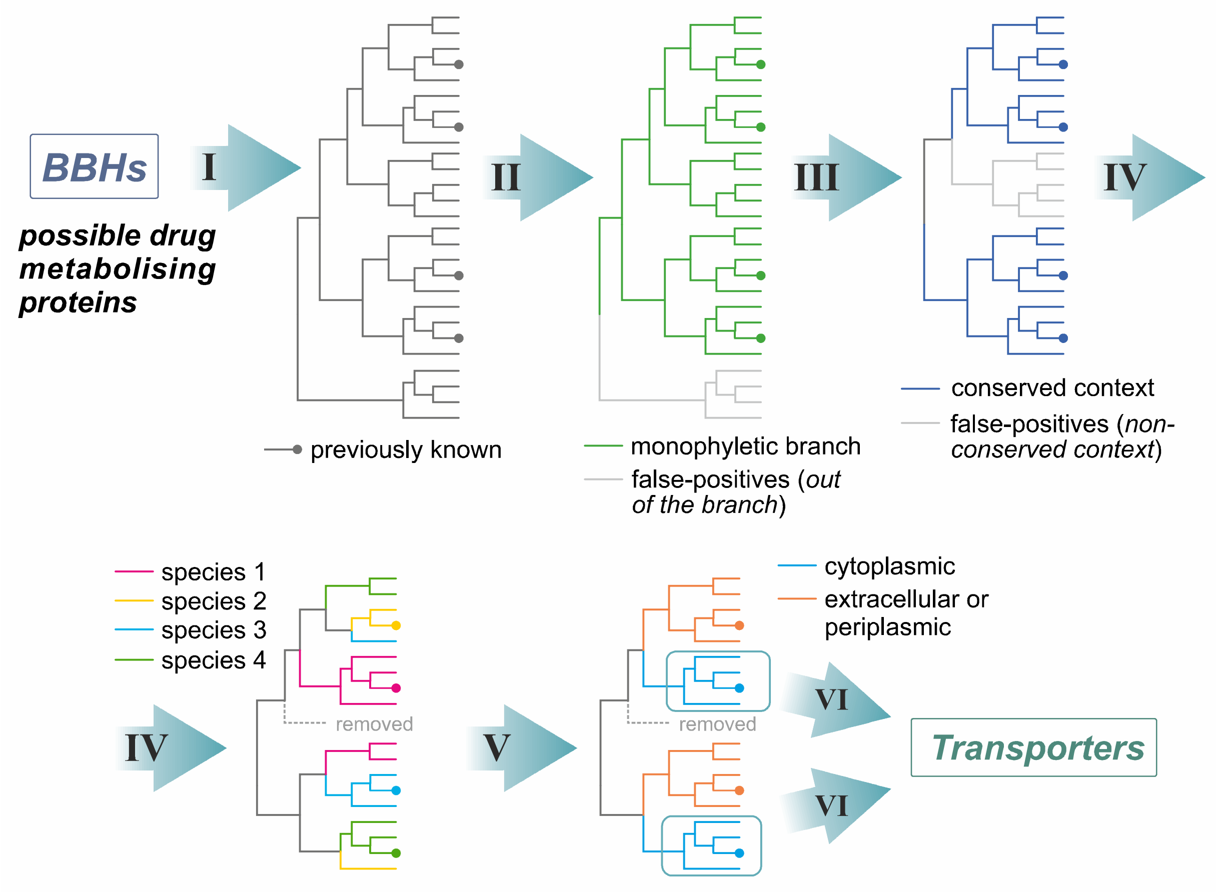


**Figure S10:** Procedures for a manual refinement of the drug-metabolising genes. The following steps are shown: (I) construction of the maximal-likelihood phylogenetic tree, rooted at a mid-point, and mapping of the previously known proteins; (II) defining monophyletic branches including all the previously known proteins; (III) removal of the false-positive predictions and analysis of the genomic context; (IV) removal of the false-positive predictions and defining of the species-specific protein clusters; (V) prediction of the subcellular localisation; (VI) analysis of the genomic context to predict transporters, only for cytoplasmic enzymes.

## Supplemental References


1. Magnusdottir, S. et al. Generation of genome-scale metabolic reconstructions for 773 members of the human gut microbiota. *Nat Biotechnol* **35**, 81-89 (2017).
2. Forster, S.C. et al. A human gut bacterial genome and culture collection for improved metagenomic analyses. *Nat Biotechnol* **37**, 186-192 (2019).
3. Qin, J. et al. A human gut microbial gene catalogue established by metagenomic sequencing. *Nature* **464**, 59-65 (2010).
4. Baldini, F. et al. Parkinson's disease-associated alterations of the gut microbiome predict disease-relevant changes in metabolic functions. *BMC Biol* **18**, 62 (2020).
5. Yachida, S. et al. Metagenomic and metabolomic analyses reveal distinct stage-specific phenotypes of the gut microbiota in colorectal cancer. *Nat Med* **25**, 968-976 (2019).
6. Lagier, J.C. et al. The rebirth of culture in microbiology through the example of culturomics to study human gut microbiota. *Clin Microbiol Rev* **28**, 237-264 (2015).
7. Poyet, M. et al. A library of human gut bacterial isolates paired with longitudinal multiomics data enables mechanistic microbiome research. *Nat Med* **25**, 1442-1452 (2019).
8. Almeida, A. et al. A new genomic blueprint of the human gut microbiota. *Nature* **568**, 499-504 (2019).
9. Pasolli, E. et al. Extensive Unexplored Human Microbiome Diversity Revealed by Over 150,000 Genomes from Metagenomes Spanning Age, Geography, and Lifestyle. *Cell* **176**, 649-662 e620 (2019).
10. Xiao, L. et al. A catalog of the mouse gut metagenome. *Nat Biotechnol* **33**, 1103-1108 (2015).
11. Heinken, A., Magnusdottir, S., Fleming, R.M.T. & Thiele, I. DEMETER: Efficient simultaneous curation of genome-scale reconstructions guided by experimental data and refined gene annotations. *Bioinformatics* (2021).
12. Tramontano, M. et al. Nutritional preferences of human gut bacteria reveal their metabolic idiosyncrasies. *Nat Microbiol* **3**, 514-522 (2018).
13. Sung, J. et al. Global metabolic interaction network of the human gut microbiota for context-specific community-scale analysis. *Nat Commun* **8**, 15393 (2017).
14. Krieg, N. et al. Bergey's Manual® of Systematic Bacteriology. (2010).
15. Griffiths, E. & Gupta, R.S. Distinctive protein signatures provide molecular markers and evidence for the monophyletic nature of the deinococcus-thermus phylum. *J Bacteriol* **186**, 3097-3107 (2004).
16. Meijer, W.G., Nienhuis-Kuiper, M.E. & Hansen, T.A. Fermentative bacteria from estuarine mud: phylogenetic position of Acidaminobacter hydrogenoformans and description of a new type of gram-negative, propionigenic bacterium as Propionibacter pelophilus gen. nov., sp. nov. *Int J Syst Bacteriol* **49 Pt 3**, 1039-1044 (1999).
17. Feist, A.M., Scholten, J.C., Palsson, B.O., Brockman, F.J. & Ideker, T. Modeling methanogenesis with a genome-scale metabolic reconstruction of Methanosarcina barkeri. *Molecular systems biology* **2**, 2006 0004 (2006).
18. van der Maaten, L. & Hinton, G. Viualizing data using t-SNE. *Journal of Machine Learning Research* **9**, 2579-2605 (2008).

19. Maini Rekdal, V., Bess, E.N., Bisanz, J.E., Turnbaugh, P.J. & Balskus, E.P. Discovery and inhibition of an interspecies gut bacterial pathway for Levodopa metabolism. *Science* **364** (2019).
20. Heinken, A. et al. Systematic assessment of secondary bile acid metabolism in gut microbes reveals distinct metabolic capabilities in inflammatory bowel disease. *Microbiome* **7**, 75 (2019).
21. Rajilic-Stojanovic, M. & de Vos, W.M. The first 1000 cultured species of the human gastrointestinal microbiota. *FEMS Microbiol. Rev.* **38**, 996-1047 (2014).
22. Wu, M. & Eisen, J.A. A simple, fast, and accurate method of phylogenomic inference. *Genome Biol.* **9**, R151 (2008).
23. Yu, C.S., Chen, Y.C., Lu, C.H. & Hwang, J.K. Prediction of protein subcellular localization. *Proteins* **64**, 643-651 (2006).
24. Yu, C.S., Lin, C.J. & Hwang, J.K. Predicting subcellular localization of proteins for Gram-negative bacteria by support vector machines based on n-peptide compositions. *Protein Sci.* **13**, 1402-1406 (2004).
25. Marchler-Bauer, A. et al. CDD: conserved domains and protein three-dimensional structure. *Nucleic Acids Research* **41**, D348-D352 (2013).
